# Single-Crystal Diamond Nanowires Embedded with Platinum Nanoparticles for High-Temperature Solar-Blind Photodetector

**DOI:** 10.1007/s40820-025-01746-9

**Published:** 2025-04-16

**Authors:** Jiaqi Lu, Xinglai Zhang, Shun Feng, Bing Yang, Ming Huang, Yubin Guo, Lingyue Weng, Nan Huang, Lusheng Liu, Xin Jiang, Dongming Sun, Huiming Cheng

**Affiliations:** 1 School of Materials Science and Engineering, University of Science and Technology of China, Shenyang, 110016 People’s Republic of China; 2 Shenyang National Laboratory for Materials Science, Institute of Metal Research, Chinese Academy of Sciences, Shenyang, 110016 People’s Republic of China; 3 School of Environment and Chemical Engineering, Shenyang University of Technology, Shenyang, 110870 People’s Republic of China; 4 Institute of Materials Engineering, University of Siegen, 57076 Siegen, Germany; 5 Institute of Technology for Carbon Neutrality, Shenzhen Institute of Advanced Technology, Chinese Academy of Sciences, Shenzhen, 518055 People’s Republic of China

**Keywords:** Diamond, Nanowire, High temperature, UV photodetector, Microwave plasma chemical vapor deposition

## Abstract

Diamond, an ultrawide-bandgap semiconductor material, is promising for solar-blind ultraviolet photodetectors in extreme environments. However, when exposed to high-temperature conditions, diamond photodetector surfaces are unavoidably terminated with oxygen, leading to low photoresponsivity. To address this limitation, single-crystalline diamond nanowires (DNWs) embedded with platinum (Pt) nanoparticles were developed using Pt film deposition followed by chemical vapor deposition (CVD) homoepitaxial growth. During the CVD, Pt nanoparticles (approximately 20 nm in diameter) undergo dewetting and become uniformly embedded within the single-crystalline DNWs. Photodetectors fabricated with these Pt nanoparticles-embedded DNWs achieve a responsivity of 68.5 A W^−1^ under 220 nm illumination at room temperature, representing an improvement of approximately 2000 times compared to oxygen-terminated bulk diamond devices. Notably, the responsivity further increases with temperature, reaching an exceptional value of 3098.7 A W^−1^ at 275 °C. This outstanding performance is attributed to the synergistic effects of the one-dimensional nanowire structure, deep-level defects, the localized surface plasmon resonance effects induced by embedded Pt nanoparticles, and localized Schottky junctions at the Pt/diamond interface, which enhance optical absorption, carrier generation, and separation efficiency. These results highlight the significant potential of Pt nanoparticles-embedded DNWs for advanced deep ultraviolet detection in harsh environments, including aerospace, industrial monitoring, and other applications.

**Supplementary Information:**

The online version contains supplementary material available at 10.1007/s40820-025-01746-9.

## Highlights

- Single-crystalline diamond nanowires embedded with platinum nanoparticles were fabricated for high-temperature solar-blind photodetection.
- At room temperature, the photodetector's responsivity represents a 2000-fold enhancement compared to bulk diamond. At 275 °C, the device demonstrates a responsivity of 3098.7 A W^-1^, while maintaining excellent spectral selectivity.
- Multiple factors synergistically enhance performance, including one-dimensional carrier transport channels, deep-level defects, localized surface plasmon resonance effect, and localized Schottky junctions.

**Supplementary Information:**

The online version contains supplementary material available at 10.1007/s40820-025-01746-9.

## Graphical abstract

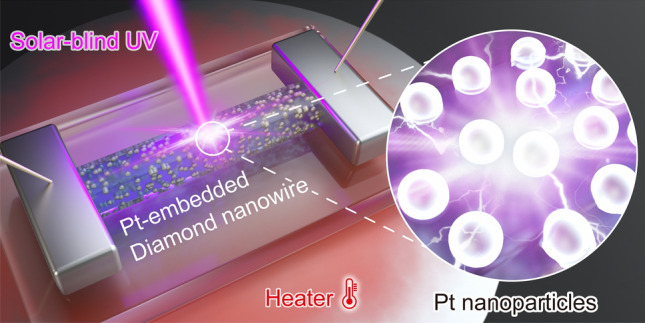

## Introduction

Ultraviolet (UV) photodetectors operating in the solar-blind region (below 280 nm) have attracted significant attention due to their unique ability to detect UV radiation without interference from sunlight [1]. This capability makes them indispensable for various critical applications, such as missile tracking, early warning systems, flame detection, ozone layer monitoring, biological research, and optical communications [2]. An ideal solar-blind UV photodetector should exhibit high spectral sensitivity and strong resistance to harsh working environments, particularly high temperatures [3, 4]. However, commercial photodetectors based on silicon (Si) semiconductor materials are limited to operating temperatures below 125 °C [5], which restricts their application in these demanding scenarios. Among ultrawide-bandgap semiconductor materials, diamond stands out due to its excellent physical and chemical properties, including an intrinsic optical absorption wavelength at 225 nm (*E*_g_ = 5.47 eV), high electrical breakdown field, excellent thermal conductivity, and the highest displacement energy (about 35 eV) among known materials [6, 7], making it a promising candidate for solar-blind UV photodetectors capable of stable operation at high temperatures in harsh environments. However, the difficulty of n-type doping has limited the development of diamond-based p–n junction UV photodetectors. As a result, planar metal–semiconductor-metal (MSM) structures are commonly used in fabricating diamond photodetectors. Numerous studies have reported that the surface termination of diamonds plays a crucial role in determining the performance of these devices [8–10]. Hydrogen and oxygen are the two most common types of surface terminations for diamonds. For hydrogen-terminated diamonds, a two-dimensional hole gas (2DHG) is formed at the near-surface layer owing to the transferred doping mechanism with adsorbed water molecules in the ambient, leading to shallow-level traps and high photocurrent gain [11–13]. However, this type of detector often exhibits a high dark current. Moreover, hydrogen bonds and water molecules desorb from the diamond surface at high temperatures, which degrades the detector’s performance at high temperatures [12, 14].

In contrast, oxygen-terminated diamond offers excellent chemical inertness and stability at high temperatures. However, its application in deep ultraviolet (DUV) detection is hindered by low responsivity, primarily due to its indirect bandgap and high density of deep-level traps on the surface that promote carrier recombination [15]. To address this challenge, researchers have explored various strategies to enhance the responsivity of oxygen-terminated diamond photodetectors. To improve the collection efficiency of photogenerated carriers, one approach involves optimizing electrode structures on single-crystal diamond surfaces [16, 17]. For example, photodetectors with three-dimensional groove-shaped electrodes have shown a 50% increase in responsivity compared to planar MSM detectors, reaching a value of 0.107 A W^−1^ [17]. Another promising strategy is surface modification with noble metal nanoparticles, which can leverage localized surface plasmon resonance (LSPR) effects, enhancing DUV absorption by increasing scattering and extending the light travel path within the diamond, thereby improving both light absorption and conversion of photogenerated carriers [18–20]. For example, coupling diamond with Pd nanoparticles resulted in a three-order enhancement in responsivity, achieving a value of 57.28 × 10^–3^ A W^−1^ [19]. Furthermore, constructing one-dimensional (1D) structures has proven effective in further enhancing responsivity [21, 22]. For diamond, ultrananocrystalline diamond nanowires (DNWs) have shown promising results with high responsivity, although their spectral selectivity still requires significant improvement [18]. Therefore, while progress has been made, further improvement of oxygen-terminated diamond-based photodetectors is essential to develop solar-blind UV detectors that can operate reliably and efficiently in harsh environments.

In this study, single-crystal DNWs embedded with Pt nanoparticles were fabricated using a process of surface Pt nanoparticle decoration followed by homoepitaxial growth of diamond. The Pt nanoparticles are uniformly distributed in a band-like pattern within the DNWs, which exhibit smooth surfaces and maintain single-crystal integrity. The Pt nanoparticles-embedded DNW with oxygen termination exhibits a 220 nm responsivity of 68.5 A W^−1^ at room temperature (RT), which is 2000 times higher than a bulk diamond device. Additionally, the UV/visible rejection ratio reaches approximately 550. Remarkably, as the operating temperature increases to 275 °C, the responsivity rises gradually and reaches 3098.7 A W^−1^. These results highlight the potential of Pt nanoparticles-embedded DNWs for high-temperature solar-blind UV photodetection applications.

## Experimental Section

### Fabrication of Pt Nanoparticles-Embedded DNWs

First, the original DNWs were transferred onto quartz substrates (SiO_2_, 15 × 15 mm^2^). The original DNWs were prepared by high-temperature etching of [100]-oriented micro-/nanocomposite diamond films, similar to the method of fabricating diamond nanoneedles reported in our previous work [23]. The preparation of the original DNWs is beyond the scope of this work. Subsequently, Pt films with thicknesses of 0.5, 1, 2, and 3 nm were deposited on the surface of the DNWs via electron beam evaporation. Finally, a microwave plasma chemical vapor deposition (MPCVD, Cyrannus 915 MHz, Iplas) system was used to dewet the Pt film and then perform diamond homoepitaxial growth. For the Pt dewetting process, pure hydrogen (H_2_) gas was flowed into the MPCVD chamber to form Pt nanoparticles on the surface of the DNWs. For the diamond homoepitaxial deposition process, the growth parameters were as follows: microwave power of 7 kW, gas pressure of 40 mbar, H_2_ flow rate of 400 sccm, methane (CH_4_) flow rate of 4 sccm, growth temperature of 750 °C, and duration of 40 min. After the homoepitaxial growth, the Pt nanoparticles were embedded within the DNWs, forming the Pt nanoparticles-embedded DNW structures (Pt-embedded DNW) with different particle sizes.

### Fabrication of the Photodetectors

Using the standard photolithography process, titanium (Ti) electrodes with a thickness of 120 nm and a spacing of 12 μm were deposited at the two ends of the Pt-embedded DNWs. After the Ti electrode deposition, the samples were annealed in an argon (Ar) atmosphere at 750 °C for 30 min to form a titanium carbide (TiC) interfacial layer between the Ti electrodes and the DNW to obtain an ohmic contact. Finally, the devices underwent oxygen plasma treatment (PIE Scientific) to transform the DNWs’ surface from hydrogen termination to oxygen termination. The parameters of oxygen plasma treatment were as follows: the power of 50 W, oxygen (O_2_) gas flow rate of 10 sccm, and treatment duration of 15 min. The devices with different Pt thicknesses of *x* were named as Pt(*x*)-embedded-DNW, where *x* denotes 0.5, 1, 2, and 3 nm. Unless otherwise specified, the Pt-embedded-DNW represents the sample with *x* = 2 nm. In control, another two types of oxygen-terminated DNWs were also used for device fabrication: the pure DNW that underwent only homoepitaxial growth, named as Pure-DNW; and the DNW subjected to homoepitaxial growth followed by Pt nanoparticles decoration, named as Pt-decorated-DNW. In addition, a device based on a single-crystal diamond (5 × 5 mm^2^) that underwent the same homoepitaxial growth process was also provided for comparison.

### Microstructure and Optoelectronic Characterization

The microstructures of the DNWs after different treatment processes were characterized using field-emission scanning electron microscopy (SEM, Hitachi SU-70) equipped with an energy dispersive X-ray spectroscopy (EDS, Horiba X-MaxN 50), transmission electron microscopy (TEM, FEI G2 F20 and FEI Titan Cube G2300), and confocal Raman spectroscopy (LabRAM HR Evolution, Horiba). The cross-sectional TEM sample was prepared by focused ion beam (FIB) milling of the quartz substrate sample. For the preparation of the planar TEM sample, the DNWs were transferred onto the surface of Molybdenum (Mo) mesh grids followed by Pt deposition and diamond homoepitaxial growth, using the same parameters as those applied on the quartz substrate. Raman spectra were collected using a 50 mW, 532 nm excitation laser with an acquisition time of 3 s. Finite-difference time-domain (FDTD) simulations were performed using FDTD Solutions software to investigate the LSPR effect of Pt nanoparticles on the optical absorption of diamond. In the simulations, one Pt nanoparticle with varying diameters (ranging from 5 to 55 nm) was placed within the diamond matrix. Two analysis groups, each consisting of six monitors, were positioned around the Pt nanoparticle to measure the scattering and absorption cross sections under light irradiation. The dark and illuminated current–voltage (*I*-*V*) characteristics, along with the time-dependent photoresponse of the devices, were measured using a semiconductor analyzer (Keithley 4200-SCS). A 1000 W xenon lamp (LEOPTICS) equipped with a monochromator provided illumination at different wavelengths, while the intensity of the incident light was measured using an optical power meter (Newport 818-UV21).

## Results and Discussion

### Microstructural Characterization of Pt-Embedded DNW

**Fig. 1:**
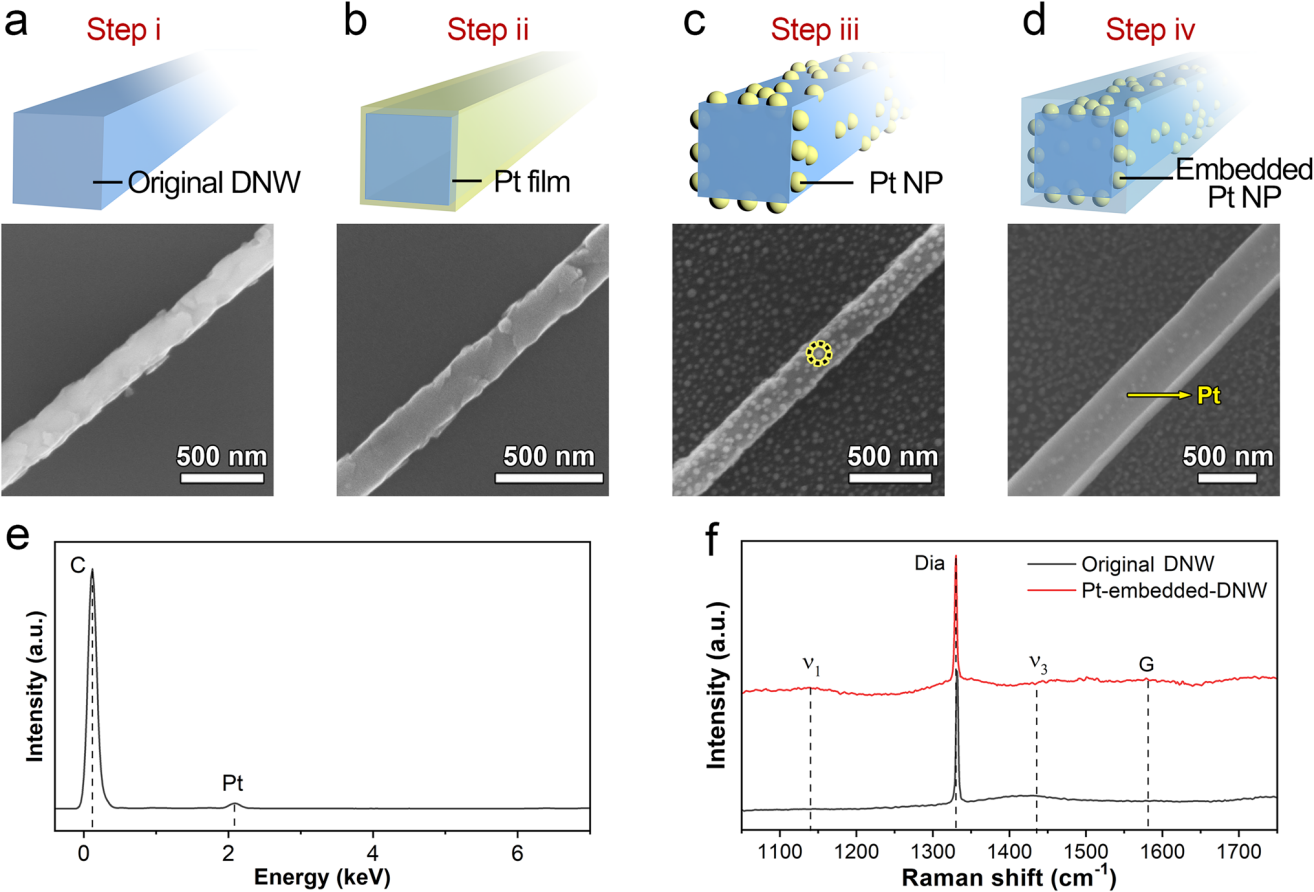
Fabrication of Pt nanoparticles-embedded DNWs by a four-step process: **a** Step i, preparation of the original DNWs by deposition of [001]-orientated micro-/nanograin diamond film and thermal oxidation; **b** Step ii, depositing a 2-nm Pt film on the surface of the DNWs; **c** Step iii, dewetting of the Pt film into nanoparticles by MPCVD; **d** Step iv: diamond homoepitaxial regrowth by MPCVD to form Pt-embedded DNWs. SEM images corresponding to each step are given in the figure; **e** EDS spectrum collected from the yellow dotted regions in **c**; **f** Raman spectra of the DNWs before and after Pt embedding and diamond homoepitaxial growth

The DNWs embedded with Pt nanoparticles were fabricated through a four-step process, as illustrated in Fig. 1a-d, with corresponding SEM images presented for each step. A sample prepared by depositing a 2-nm-thick Pt film was used as an example. In the first step (i), DNWs were prepared by selectively etching the nanocrystalline diamond phase in a [100]-oriented micro-/nanocomposite diamond film [23]. As shown in Figs. 1a and S1, the original DNWs feature a diameter of approximately 250 nm and a length of 16 μm. In the second step (ii), the Pt film was deposited on the DNW surfaces using electron beam evaporation (Fig. 1b). Due to the continuous coverage, the Pt film is challenging to distinguish at this stage. In the third step (iii), the sample was transferred into the MPCVD chamber, where the Pt film underwent dewetting in a pure hydrogen gas plasma environment, forming Pt nanoparticles with an average diameter of approximately 20 nm uniformly distributed on the diamond surface (Fig. 1c). In the final step (iv), homoepitaxial regrowth of diamond was carried out in a hydrogen/methane mixed gas plasma to form Pt-embedded DNWs (Fig. 1d). During this process, an epitaxial layer was deposited on the surface of the Pt nanoparticles-decorated DNWs. As shown in Fig. 1d, the resulting nanowires exhibit smooth surfaces and an increased diameter of about 400 nm, with the Pt nanoparticles faintly visible in the SEM image.

The EDS spectrum collected from the yellow dotted regions in Fig. 1c reveals the presence of Pt and C elements (Fig. 1e), providing clear evidence of the formation of Pt nanoparticles. Figure 1f presents the Raman spectra of a single DNW before and after homoepitaxial growth. Both spectra exhibit a sharp peak at 1332 cm^−1^, attributing to the *T*_2g_ signature of diamond [24]. The full width at half maximum (FWHM) of the Raman peak, an indicator of crystallization quality, increases from 2.71 cm^−1^ for the original DNW to 3.38 cm^−1^ for the Pt nanoparticles-embedded DNW, which implies that the crystalline quality of the epitaxial layer on the DNW slightly decreases. For the original DNW, the absence of D and G bands confirms its high crystallinity [25]. A trans-polyacetylene (TPA) peak (*v*_3_) centered at 1420 cm^−1^ indicates the presence of small-sized diamond grains, which originate from the nanoscale protrusions on the diamond surface [26]. For the Pt nanoparticles-embedded DNW, the Raman peaks, including the *v*_1_ band at 1140 cm^−1^, the G band at 1580 cm^−1^, and the *v*_3_ band, are weak, indicating that the Pt-embedded DNWs maintain good crystalline quality.

**Fig. 2:**
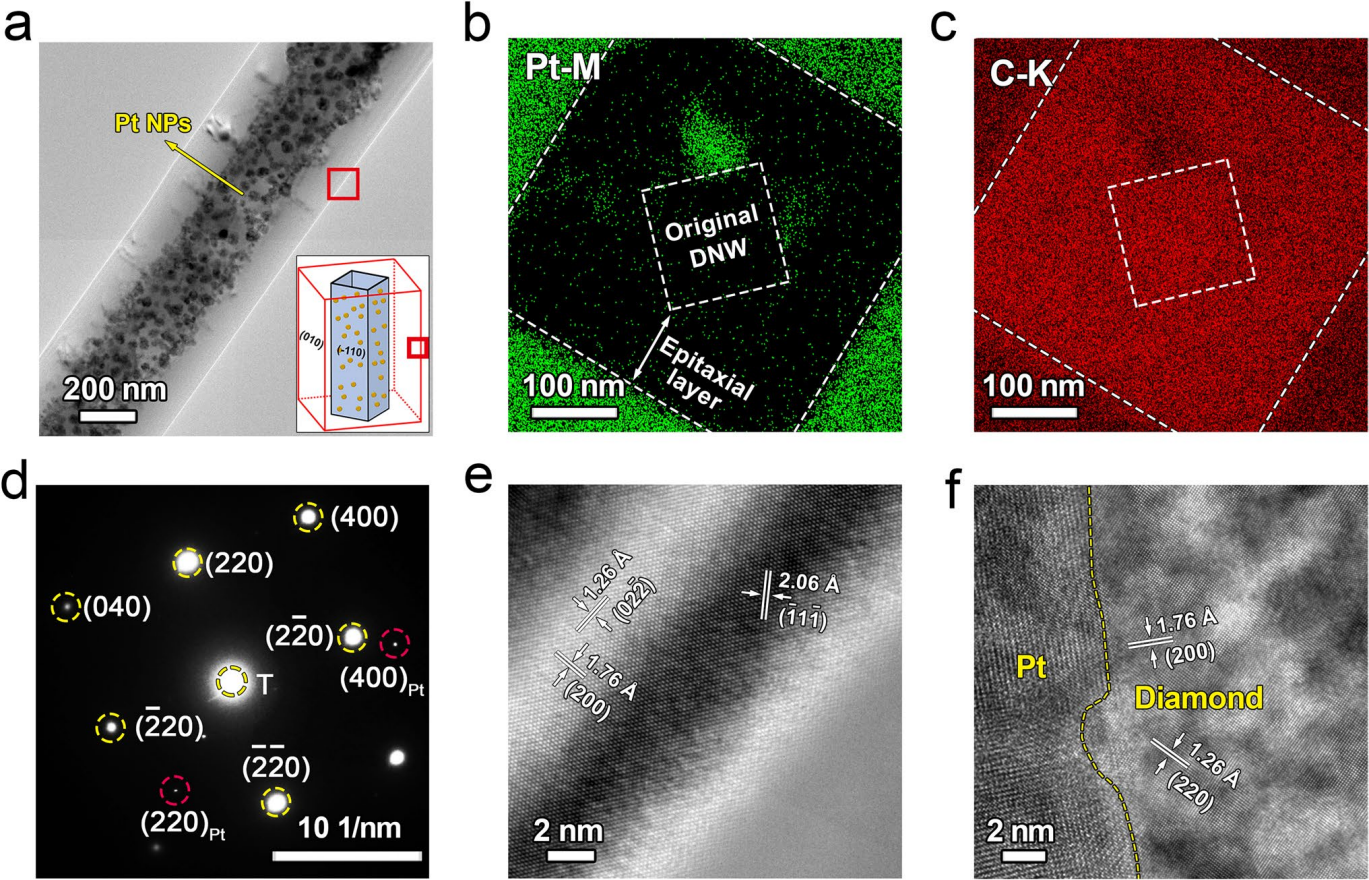
Microstructural characterization of a DNW embedded with Pt nanoparticles: **a** Low-magnification TEM image. The inset illustrates the Pt distribution between the original nanowire and the homoepitaxial diamond layer; **b-d** elemental mappings of Pt–M, C–K, and selected area electron diffraction pattern obtained from the cross section of the nanowire, respectively; **e** HRTEM image of the epitaxial diamond layer taken from [011] zone axis; **f** HRTEM image of the Pt/diamond interface taken from the [001] zone axis

Figure 2a shows low-magnification TEM images of a Pt nanoparticles-embedded DNW obtained from the 2-nm-thick Pt film sample. The nanowire exhibits a smooth surface, with an average diameter of ~ 370 nm, slightly smaller than that observed in the SEM images. Numerous Pt nanoparticles, visible as dark contrast within the DNW, display a band-like distribution along the length of the nanowire. Statistical analysis reveals an average particle size of approximately 20 nm (Fig. S2f). In comparison, the TEM images were also acquired from the other Pt nanoparticles-embedded DNWs with different Pt film thicknesses (Fig. S2a-c). All the nanowires exhibit smooth surfaces with uniform distributions of Pt nanoparticles. As the thickness of the Pt films increases from 0.5 to 1, 2, and 3 nm, the average size of Pt nanoparticles increases gradually from 6 to 10, 20, and 45 nm, respectively, based on statistical analysis (Fig. S2d-g). This implies that the size of Pt nanoparticles embedded in diamond could be tailored by the thickness of Pt films.

In order to characterize the distribution of Pt nanoparticles, a cross-sectional TEM sample of the Pt-embedded DNW was prepared using FIB milling. EDS mapping was employed to characterize the chemical and the interface between the original DNW and the homoepitaxial layer (Fig. 2b, c). The interface can be distinguished by the location of the Pt nanoparticles (Fig. S3a). In Fig. 2b, the Pt-M mapping indicates that the inner dotted rectangle corresponds to the original DNW, while the outer dotted rectangle corresponds to the cross-sectional morphology of the Pt-embedded DNW. The presence of Pt beyond the outer rectangle is attributed to additional Pt deposition during the FIB preparation. The results reveal that the Pt nanoparticles are embedded at the interface between the original DNW and the epitaxial layer. The selected area electron diffraction (SAED) pattern, taken from the cross-sectional TEM sample, indicates that the DNW remains single crystalline after epitaxial growth (Fig. 2d). The diffraction spots corresponding to the Pt lattice confirm the embedding of Pt grains within the single-crystal diamond lattice. Additionally, the four sidewalls of the regrown nanowire are indexed to the {100} crystal planes of the cubic diamond phase. A high-resolution TEM (HRTEM) image of the epitaxial layer (Fig. 2e) was captured from the region marked by red boxes in Fig. 2a. To obtain a high-quality HRTEM image, the sample was tilted toward the diamond [011] zone axis, as the thickness of the nanowire along the [001] axis was too large for optimal imaging. The measured interplanar spacings of 1.26, 1.76, and 2.06 Å correspond to the (02–2), (200), and (-11–1) planes of the cubic diamond lattice, respectively. The epitaxial layer exhibits high crystalline quality with the absence of crystalline defects. The sidewall plane at the tilting direction is indexed as the {110} crystalline plane, corresponding to the intersection line of the two {100} crystalline planes, consistent with the SAED results in Fig. 2d. The growth mechanism of the epitaxial layer is illustrated in the inset of Figs. 2a and S3b. Considering that the {110} plane has a lower atomic packing density and interplanar spacing than the {100} plane, the {100} plane exhibits a smaller growth rate and becomes the exposed surface after CVD growth. Owing to the small diameter and the rough surface of the original DNW, the reactive gas plasma can penetrate the interface between the DNW and substrate. Consequently, all the side surfaces including the down-side surface exhibit epitaxial diamond growth. Eventually, the HRTEM image of the Pt/diamond interface, taken along the diamond [001] zone axis from the cross-sectional sample, is shown in Fig. 2f. The interplanar spacings of 1.26 and 1.76 Å correspond to the (220) and (200) planes of cubic diamond, respectively. The Pt nanoparticle, located in the left region, overlaps with the diamond lattice, making it difficult to measure the interplanar spacings of the Pt lattice. It is noted that the epitaxially grown side of the DNW remains single crystalline. As a result, the Pt-embedded DNW exhibits good crystal quality with a uniform distribution of Pt nanoparticles, confirming the success of the fabrication process.

### Influence of Pt Nanoparticle Size on LSPR

**Fig. 3:**
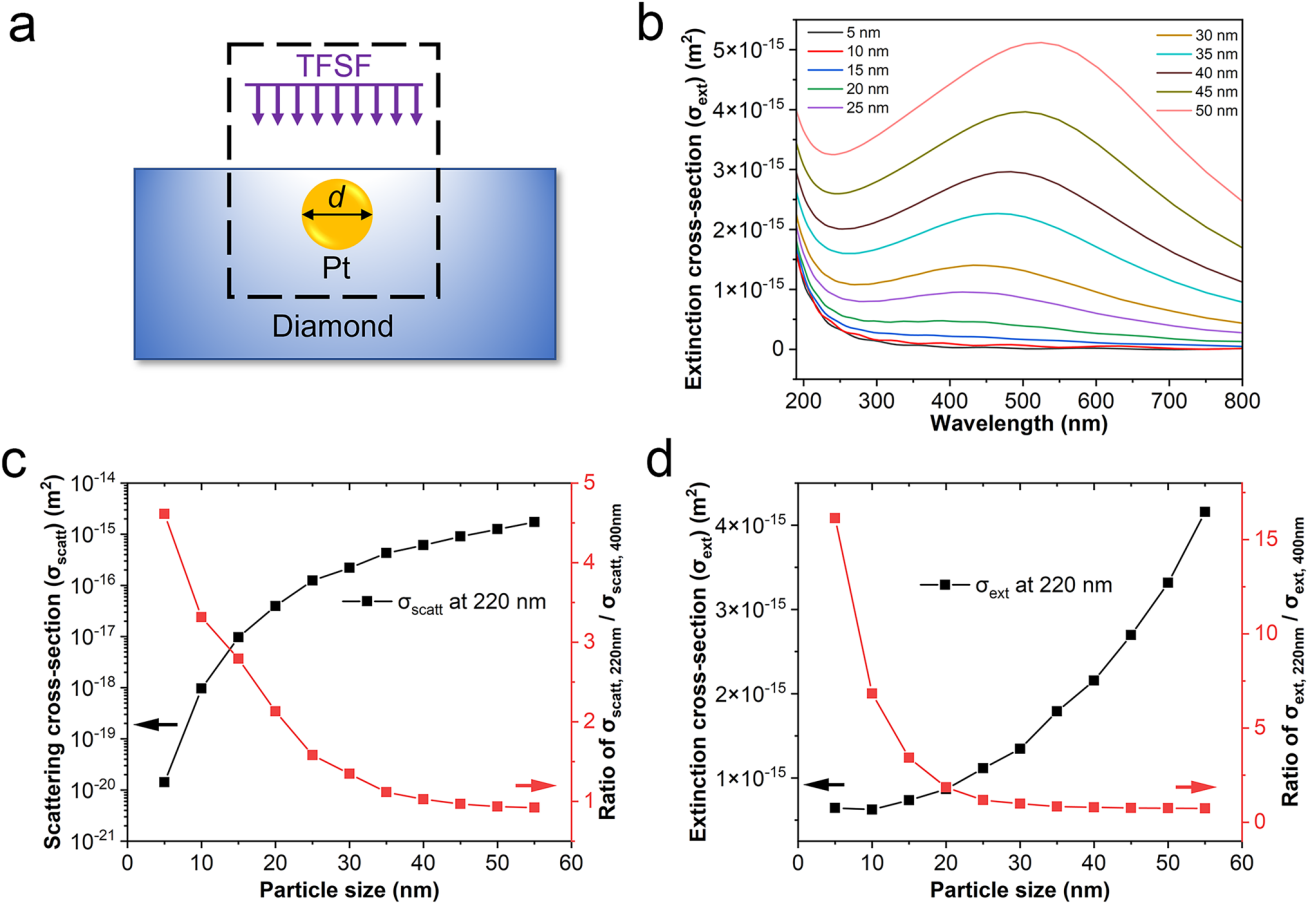
**a** Schematic diagram of the model used for FDTD simulations; **b** spectral extinction cross sections for different Pt nanoparticle sizes; **c** scattering cross section and **d** extinction cross section as a function of Pt nanoparticle size: the black curve represents the cross sections at 220 nm, and the red curve represents the ratio of the cross sections at 220 nm to that at 400 nm

Numerical simulations using the FDTD method were performed to analyze the LSPR behavior of the embedded Pt nanoparticles with varying sizes (Fig. 3). A single Pt nanoparticle was placed on the diamond surface (Fig. S4) and inside the diamond (Fig. 3a) and was illuminated using a total-field scattered-field (TFSF) source. Two analysis groups, one slightly larger and one slightly smaller than the TFSF region, were used to calculate the scattering cross section (σ_scatt_) and absorption cross section (σ_abs_). The extinction cross section (σ_ext_) was calculated using the equation $\left|{\sigma }_{\text{ext}}\right|=\left|{\sigma }_{\text{scatt}}\right|+\left|{\sigma }_{\text{abs}}\right|$. These parameters reflect the optical absorption or the LSPR effect induced by the Pt nanoparticles. It is found that the embedded Pt nanoparticles exhibit significantly larger scattering cross sections than the Pt nanoparticle on the surface at the same diameter (Fig. S4b), indicating the embedding of Pt nanoparticles inside the diamond can enhance the LSPR effect. For the Pt embedding, the LSPR peak redshifts with increasing Pt nanoparticle size (Fig. 3b). When the size of Pt nanoparticles is smaller than 20 nm, the LSPR peak lies within the DUV wavelength range, and no visible LSPR peak is observed. Both the scattering and extinction cross sections increase with the size of Pt nanoparticles (Fig. 3c, d). When the size of the Pt nanoparticles exceeds 20 nm, the strongest LSPR peak appears in the visible wavelength range in addition to the DUV peak. The scattering and extinction cross sections further increase with the Pt nanoparticle size. Consequently, the ratio of the scattering cross sections at 220 nm to that at 400 nm thus decreases with increasing Pt nanoparticle size. The extinction cross section exhibits a similar trend. This implies that a strong visible response occurs when the size of the Pt nanoparticles is greater than 20 nm, which may significantly degrade the spectral response of the device. Therefore, the optimized size of the embedded Pt nanoparticles is approximately 20 nm to achieve a strong DUV response and enhanced spectral selectivity.

### Room Temperature DUV Photoresponse Properties

**Fig. 4:**
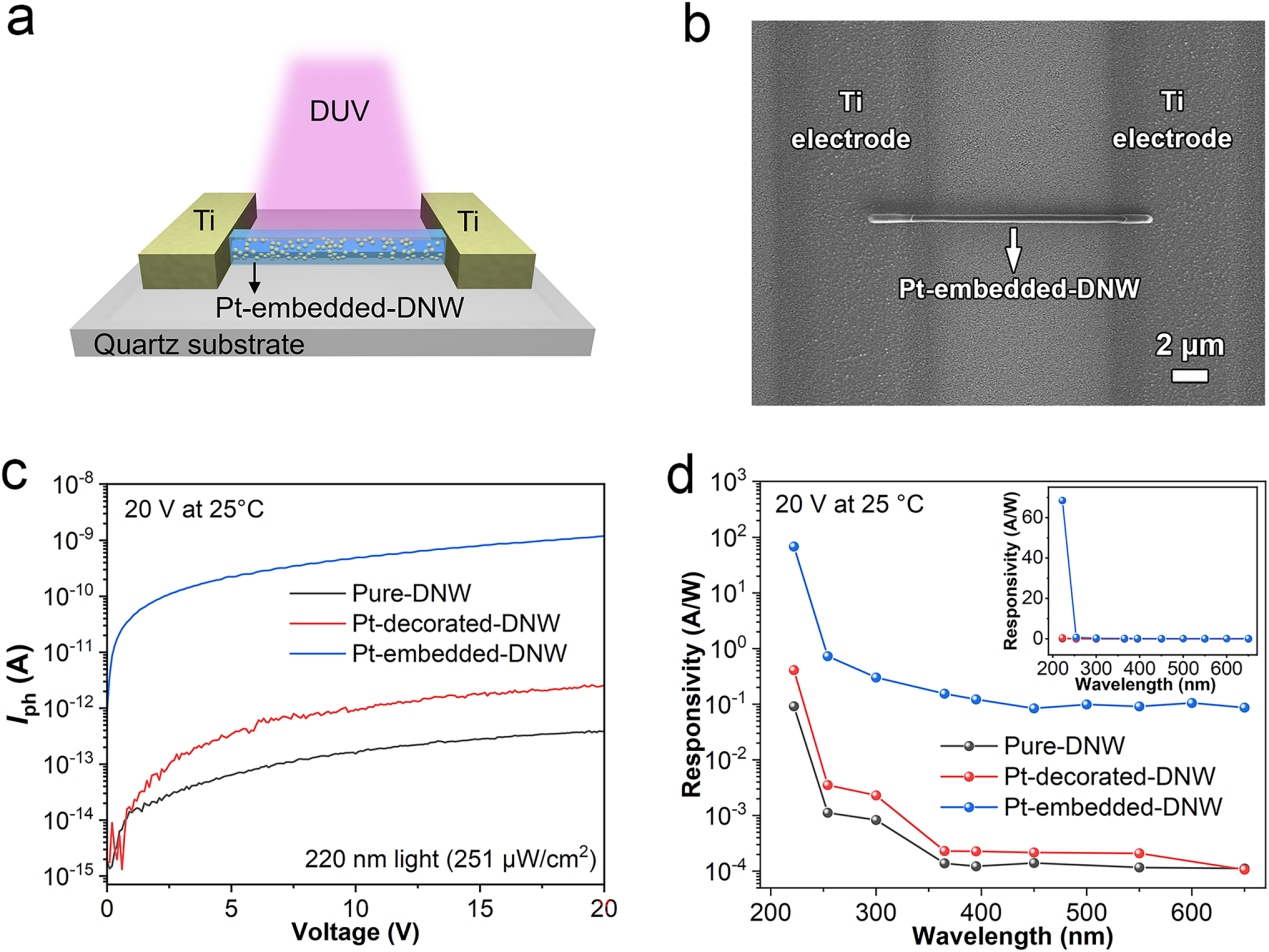
Room temperature performance of a DUV photodetector based on a Pt-embedded DNW: **a** Schematic of the device; **b** corresponding SEM image; **c** net photocurrent (*I*_ph_) versus bias voltage under 220 nm light illumination; **d** spectral responsivity at a bias of 20 V. For comparison, similar device structures were fabricated based on a pure DNW and Pt nanoparticles-decorated DNW

Figure 4a, b shows the illustration and corresponding SEM image of a DUV photodetector fabricated from Pt-embedded DNW. The size of Pt nanoparticles was chosen to be 20 nm, corresponding to the sample obtained from the 2-nm-thick Pt film. For comparison, DUV photodetectors based on pure DNW and Pt nanoparticles-decorated DNW with the same Pt film thickness were also fabricated (Fig. S5a). Figure S5b, c shows the *I-V* curves of the two photodetectors under dark and 220 nm light illumination. All the *I-V* curves under dark exhibit nearly linear behavior, indicating Ohmic contact between diamond and Ti electrodes. Figure 4c illustrates the variation of net photocurrent *I*_ph_ (*I*_ph_ = *I*_λ_—*I*_Dark_, where* I*_λ_ and *I*_Dark_ are photocurrent and dark current) with bias voltage for the three DUV photodetectors based on different DNWs. For the oxygen-terminated pure DNW (Pure-DNW), the dark current is about 0.06 pA at 20 V, while the net photocurrent reaches 0.46 pA under 220 nm light illumination. The responsivity (*R*_λ_) can be calculated using the equation: ${R}_{\lambda }=\frac{{I}_{\text{ph}}}{{P}_{\lambda }\times S}$, where *S* is the effective light area of the DNW and ${P}_{\lambda }$ is the light power intensity at wavelength of *λ*. The effective light area of different devices is shown in Table S1. The *R*_λ_ of the Pure-DNW is calculated to be 0.092 A W^−1^, which is about 2.7 times higher than that of bulk single-crystal diamond with oxygen-terminated surface (0.034 A W^−1^, Fig. S6). For the oxygen-terminated Pt nanoparticles-decorated DNW (Pt-decorated-DNW), the dark current and the net photocurrent under 220 nm light at 20 V are 0.19 and 2.06 pA, respectively. The slightly higher dark current is attributed to the introduction of Pt nanoparticles on diamond surface compared with the Pure-DNW device. The responsivity of the Pt-decorated-DNW reaches 0.41 A W^−1^, representing a 12 times enhancement compared to the Pure-DNW, which is attributed to the DUV absorption enhancement by the LSPR effect induced by Pt nanoparticles. It is worth mentioning that the large number of deep-level traps on oxygen-terminated diamond surfaces inhibit the increase of photocurrent, thereby weakening the LSPR-induced performance improvement [15, 27]. Both devices exhibit two distinct states upon light switching (on/off), demonstrating excellent stability and reproducibility for the DUV response (Fig. S7a). The on–off ratios (*I*_220 nm_/*I*_Dark_) of the Pure-DNW and the Pt-decorated-DNW are 9.6 and 10.3, respectively. The detectivity (${D}^{*}$) and external quantum efficiency (EQE) were calculated using the following equations [28]:

1 $${D}^{*}=\frac{{R}_{\lambda }}{{(2e{I}_{\text{Dark}}/S)}^{1/2}}$$

2 $$\text{EQE}=\frac{hc}{e\lambda }{R}_{\lambda }$$

where *e* is the electron charge, *h* is the Planck constant, and *c* is the speed of light. The ${D}^{*}$ of the Pure-DNW device and the Pt-decorated-DNW device is calculated to be 9.96 × 10^10^ Jones and 2.18 × 10^11^ Jones. The EQE of Pure-DNW and Pt-decorated-DNW is calculated to be about 51% and 231%, respectively. Although the Pt-decorated-DNW exhibits a substantial increase in DUV detection performance and achieves a relatively high responsivity value compared to oxygen-terminated bulk diamond, the photocurrent remains relatively low. This limitation may affect its practicality for fast and precise measurements.

For the Pt-embedded-DNW, the net photocurrent increases significantly with voltage (blue curve in Fig. 4c), reaching 1028 pA under 220 nm UV illumination at a bias of 20 V. The responsivity is calculated to be 68.5 A W^−1^, which represents a remarkable three-order-of-magnitude increase in responsivity compared to the Pure-DNW and a two-order-of-magnitude increase compared to the Pt-decorated-DNW. Such significant enhancement in photoresponsivity is attributed to the LSPR effect induced by Pt nanoparticles and the Pt/diamond Schottky junction, which will be discussed in the later section. The ${D}^{*}$ and EQE of Pt-embedded-DNW were calculated to be approximately 6.25 × 10^11^ Jones and 3.85 × 10^4^%. Additionally, the Pt-embedded-DNW exhibits good spectral selectivity with an ultraviolet to visible rejection ratio (*R*_220 nm_/*R*_visible_) of 550 (Fig. 4d), underscoring its strong preference for DUV detection. The device shows two distinct states when the 220 nm light illumination is switched on/off (Fig. S7b). The lower *I*_220 nm_/*I*_Dark_ is due to the increase in the dark current compared with the Pure-DNW and the Pt-decorated-DNW devices. Therefore, a high DUV response with excellent stability and reproducibility confirms the reliable and repeatable performance of the Pt-embedded-DNW photodetector.

Moreover, the DUV response of the Pt(*x* nm)-embedded-DNW photodetectors with different Pt nanoparticle sizes (6, 10, 20, and 45 nm) was examined (Fig. S8). The responsivity of these four devices falls within the range of 10^1^–10^2^ A W^−1^ (Fig. S8e). As the Pt nanoparticle size increases, the responsivity first rises from 12.6 A W^−1^ (6 nm sample) to 68.5 A W^−1^ (20 nm sample) and then decreases to 44.7 A W^−1^ (45 nm sample). Therefore, the 20 nm sample exhibits the highest responsivity among the four Pt-embedded-DNW devices. Additionally, the *R*_220 nm_/*R*_visible_ gradually decreases from 641 for the 6 nm sample to 370 for the 45 nm sample. This trend is consistent with the FDTD simulation results, which show that increasing the Pt nanoparticle size shifts the LSPR peak into the visible wavelength range. Therefore, the 20 nm sample demonstrates the optimized performance for DUV detection.

### High-Temperature DUV Photoresponse Properties

**Fig. 5:**
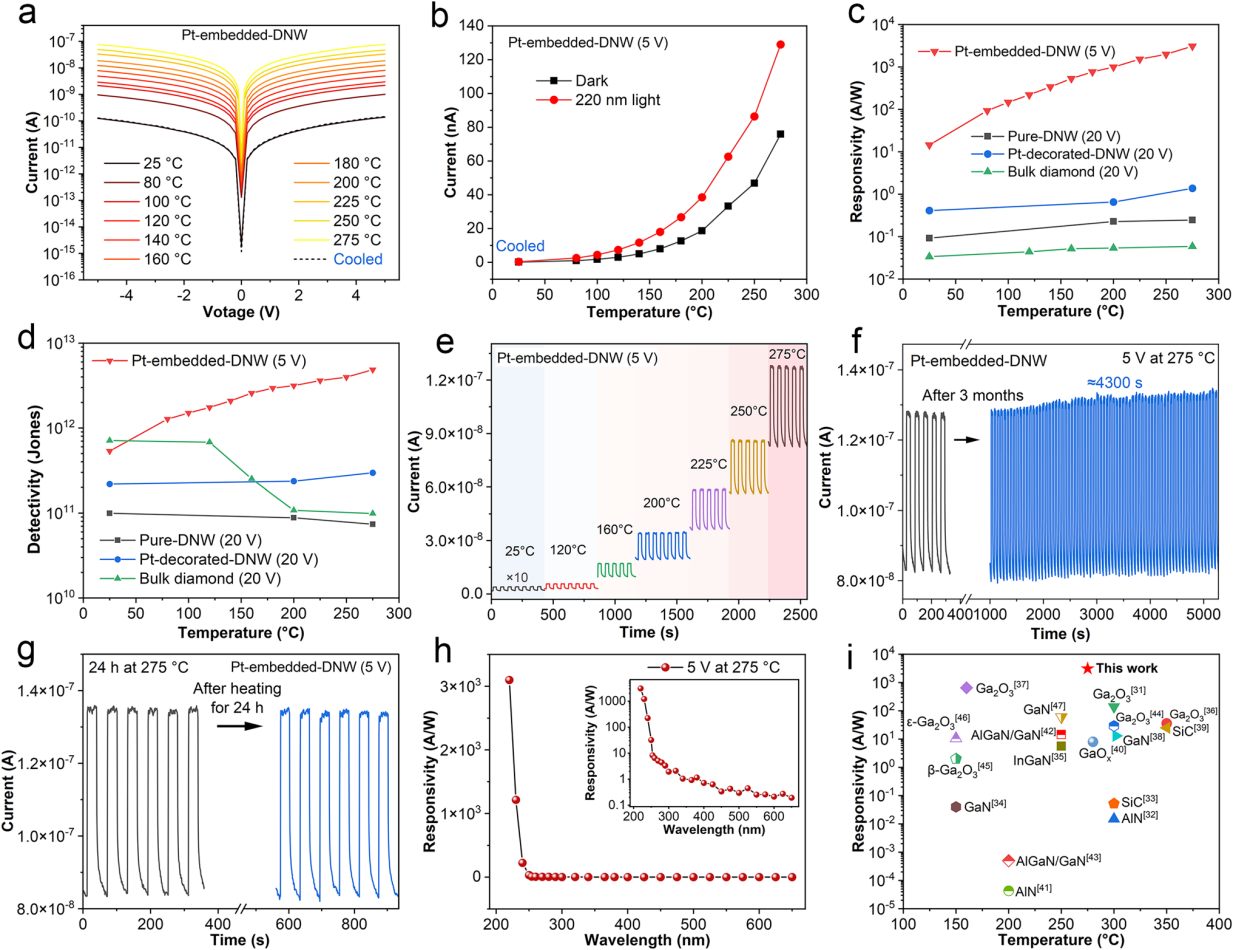
Photoelectric response of the DUV photodetectors with or without 20 nm-sized Pt nanoparticles at different temperatures ranging from 25 to 275 °C: **a** *I*-*V* curve under dark conditions of the Pt-embedded-DNW; **b** variation of dark current and photocurrent with temperature of the Pt-embedded-DNW; **c** variation of 220 nm light responsivity of different photodetectors with temperature; **d** variation of detectivity of different photodetectors with temperature; **e** time-dependent response at different temperatures and **f, g** stability evaluation at 275 °C of the Pt-embedded-DNW under 220 nm light illumination; **h** spectral response of the Pt-embedded-DNW at 275 °C; **i** comparison of photoresponsivity with other devices in previous references

The photodetector performance of the Pt-embedded-DNW with 20 nm-sized Pt nanoparticles was measured at elevated temperatures from RT to 275 °C. The bias was reduced to 5 V in order to prevent excessive current from flowing through the DNW at high temperatures. As shown in Figs. 5a and S9a, the *I*-*V* curves in dark conditions remain linear at different temperatures, suggesting Ohmic contact between the Ti electrodes and the DNW. Both the dark current and photocurrent exhibit an increase by about two orders of magnitude as the temperature rises from RT to 275 °C (Fig. 5b). Correspondingly, the *I*_220 nm_/*I*_Dark_ ratio decreases slightly from 2.6 to 1.7 (Fig. S9b). But the photoresponsivity increases gradually from 14.5 A W^−1^ at RT to an exceptional 3098.7 A W^−1^ at 275 °C (Fig. 5c). The ${D}^{*}$ also increases significantly with increasing temperature, rising from 5.35 × 10^11^ Jones at RT to 4.87 × 10^12^ Jones at 275 °C (Fig. 5d). And the EQE increases from 8.12 × 10^3^% to 1.74 × 10^6^% (Fig. S9c). In comparison, other photodetectors including bulk single-crystal diamond, Pure-DNW, and Pt-decorated-DNW were tested at a bias of 20 V. For bulk single-crystal diamond, the dark current increases by two orders of magnitude while the photocurrent increases by less than 2 times as the temperature rises from RT to 275 °C (Fig. S10a, b). The *I*_220 nm_/*I*_Dark_ ratio decreases significantly from 1201.6 to 14.3 with increasing temperature. The responsivity slightly increases to 0.059 A W^−1^, while the ${D}^{*}$ decreases significantly to 9.90 × 10^10^ Jones at 275 °C. These two values are lower than those of the Pt-embedded-DNW. For the Pure-DNW and Pt-decorated-DNW devices, the responsivity and the ${D}^{*}$ show slight variation with temperature (Fig. 5c, d). At all the temperatures, the responsivity and the ${D}^{*}$ of the Pt-decorated-DNW are higher than the corresponding values of the Pure-DNW, but substantially lower than those of the Pt-embedded-DNW. These results demonstrate that the Pt-embedded-DNW photodetector not only exhibits excellent stability at high temperatures but also achieves excellent DUV detection capabilities as the temperature increases.

Figure 5e illustrates the time-resolved photoresponses of the Pt-embedded-DNW photodetector under 220 nm light at a bias of 5 V at different temperatures. It is noted that the device exhibits stable responses to DUV light at elevated temperatures. The rise time (*t*_rise_) and decay time (*t*_decay_) are defined as the times required for the current to change from 10% to 90% and from 90% to 10% of its maximum value, respectively, when illumination starts or ends. As the temperature increases from RT to 275 °C, the* t*_rise_ of the Pt-embedded-DNW decreases from 1.26 to 0.92 s, while *t*_decay_ decreases from 9.01 to 7.09 s (Fig. S9d). The reduction in the response time is caused by the increase in carrier mobility. Generally, the carrier mobility decreases owing to the increased acoustic phonon scattering at elevated temperature. In our work, the carrier mobility at the 220 nm light illumination was measured based on a Pt-embedded-DNW field-effect transistor (Fig. S11). The carrier mobility exhibits an increase with temperature, similar to that in heavily boron-doped diamond materials [29]. The DNW at the light illumination can be regarded as being in a heavily doped state, in which ionized impurities scattering dominates over the acoustic phonon scattering, leading to the increased carrier mobility at elevated temperature. In other words, the carriers can escape from traps and participate in electric conduction as the temperature rises [30]. This contributes to the increased carrier mobility and the decreased response time of the device. In order to improve the response speed, the Pt-embedded-DNW photodetector is irradiated with periodic 220 nm DUV light and continuous 365 nm UV light (Fig. S12). The response time of *t*_rise_ and *t*_decay_ decreases to 0.76 and 4.03 s, respectively. The device demonstrates an improved response speed while maintaining no significant degradation in responsivity. Additionally, the Pt-embedded-DNW, after being operated at 275 °C, is re-tested after three months of atmospheric storage (Fig. 5f). It is noted that the device maintains a stable response to periodic 220 nm light illumination for up to 70 min at 275 °C. And the 220 nm response to illumination shows no significant degradation after undergoing 24-h thermal treatment at 275 °C (Fig. 5g). The other oxygen-terminated diamond devices also demonstrate stable responses to 220 nm light illumination at 275 °C (Fig. S10c-e). These results demonstrate that the Pt-embedded-DNW device exhibits long-term performance stability at high temperatures. The spectral responsivity with different wavelengths was also measured at 275 °C (Fig. 5h). The device exhibits a peak responsivity at 220 nm with the UV/visible rejection ratio of 4303 at 275 °C, indicating that the device still maintains good spectral selectivity for DUV illumination at high temperatures. Figure 5i compares the performance of the Pt-embedded-DNW device with the previously reported high-temperature DUV photodetectors [31–47]. Remarkably, the Pt-embedded-DNW photodetector shows a high responsivity to DUV light that does not decrease with increasing temperatures.

### Mechanism of Photoresponse Enhancement

**Fig. 6:**
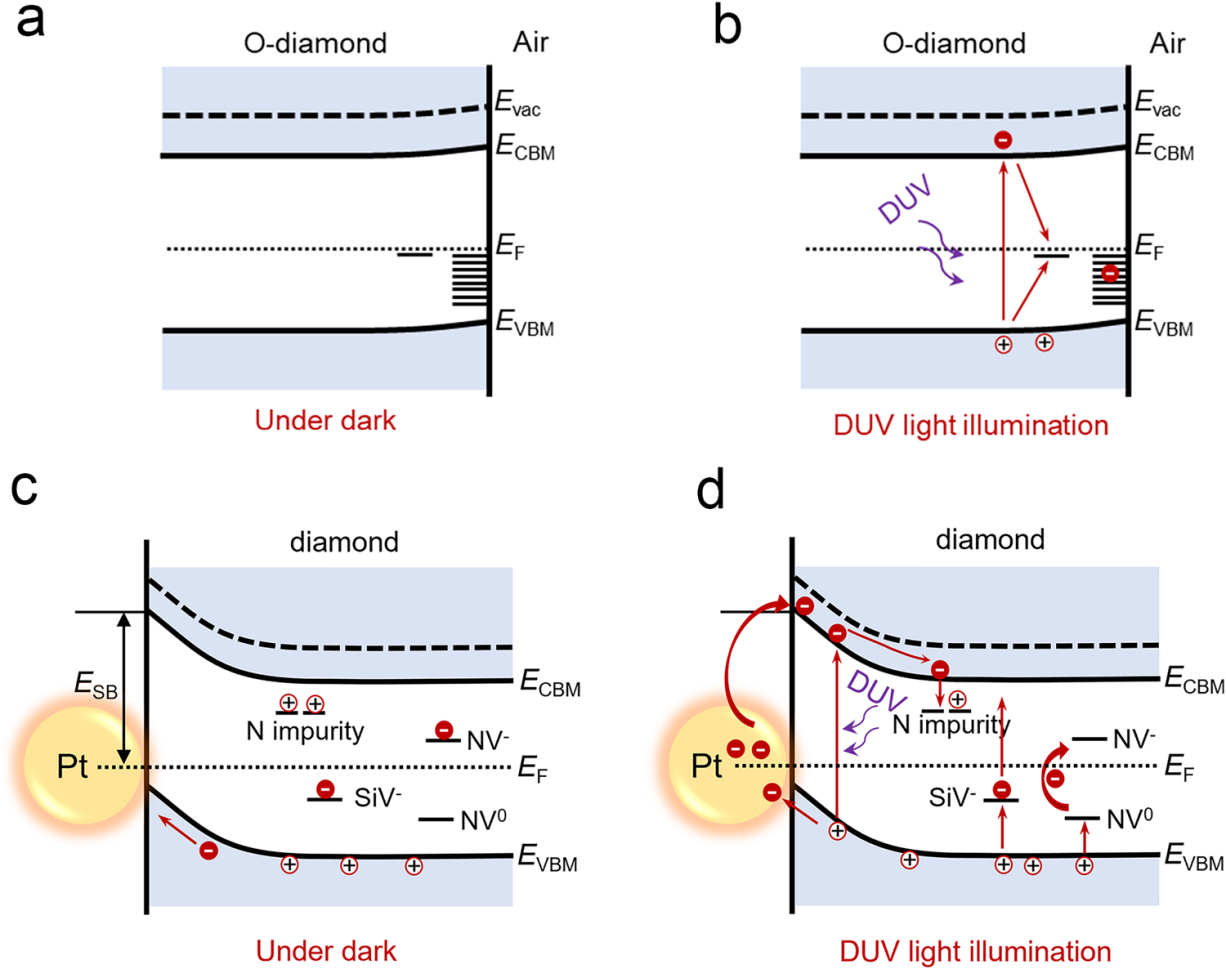
Energy band diagrams illustrating the photoresponse mechanism: Schematics of the oxygen-diamond/air interface of the Pure-DNW under dark **a**, DUV light illumination **b**. Schematics of the embedded Pt/diamond interface within the Pt-embedded-DNW under dark **c** and DUV light illumination **d**

The enhancement mechanisms of photoresponse in the DNWs with different surface modifications of Pt nanoparticles can be understood in terms of band structure. For the Pure-DNW, the responsivity is higher than that of a bulk diamond device. This improvement is attributed to the 1D directional transport of photo-induced carriers along the nanowire [22, 48–51]. However, this improvement remains limited due to a high density of surface trap states introduced by the oxygen-terminated surface (Fig. 6a). These surface traps act as deep-level defects within the bandgap, located approximately 2.0 to 2.4 eV above the valence band [15]. Under 220 nm light illumination (Fig. 6b), photogenerated electrons and holes are recombined at the surface traps, drastically reducing the number of carriers reaching electrodes and thus suppressing photoresponsivity [27]. Because of the nanowire’s large specific surface area, carrier recombination at the surface has a much stronger suppressing effect on the DNW’s photoresponse than it would in bulk diamond. Consequently, the Pure-DNW exhibits limited enhancement of the responsivity despite its structural advantages.

For the Pt-decorated-DNW and the Pt-embedded-DNW, the nanowires exhibit a single-crystal structure. However, the photoluminescence (PL) spectrum reveals the presence of nitrogen-vacancy (NV) and silicon-vacancy (SiV) centers are present in the nanowire (Fig. S13) [52]. This indicates that the impurities of N and Si are incorporated into the diamond lattice. The N impurities and NV centers are located > 1.7 eV below the conduction band minimum, while the SiV centers are located > 1.5 eV above the valence band maximum [53, 54]. These defects, as deep-level defects at room temperature, increase the dark current of the device owing to thermally assisted carrier excitation. At high temperatures, carriers captured in the traps are more easily released through thermal excitation, thereby exhibiting dynamic characteristics similar to shallow-level traps, which continuously capture and release carriers, thereby extending the carrier lifetime and increasing the photogain. The net photocurrent of the Pt-embedded-DNW was measured under different light power intensity of the 220 nm illumination at RT and 200 °C (Fig. S14a, b). The net photocurrent can be expressed as ${I}_{\text{ph}}\propto {P}^{0.3}$ at RT and ${I}_{\text{ph}}\propto {P}^{0.24}$ at 200 °C. At both temperatures, the exponent of < 1 reflects that the photo-induced carriers undergo multiple recombination or trapping during the transport process [3, 55]. The deep-level defects are proposed to function as deep-level traps at room RT while acting as shallow-level traps under high-temperature conditions.

In addition, numerous Schottky junctions are formed at the Pt/diamond interface due to the difference in their work functions (5.65 eV for Pt and about 4 ~ 5 eV for oxygen-terminated diamond) [11], as shown in Fig. 6c. Electron transfer between diamond and Pt after their contact leaves behind a depletion region in diamond and causes upward band bending of the diamond's energy levels. Photo-induced carriers can be separated efficiently in the depletion region under the built-in electric field, which increases the photocurrent collected by the electrodes. Furthermore, a portion of the DUV light is directly absorbed by Pt nanoparticles, generating hot electrons that cross the Schottky barrier into the conduction band of diamond, thereby contributing to the photocurrent. Thirdly, the 20 nm-sized Pt nanoparticles induce LSPR effect under the 220 nm light illumination, as revealed by FDTD simulations, which generates a locally intensified electric field. This enhanced electric field increases the effective light absorption at the Pt/diamond interface, thereby improving the photoresponse. Compared to the Pt-decorated-DNW, the Pt-embedded-DNW has a larger Pt/diamond interfacial contact area, which simultaneously produces a stronger LSPR effect (Fig. S4) and forms more Pt/diamond Schottky junctions. These features collectively contribute to the increased photoresponse of the device.

Based on the above discussion, the enhanced responsivity of the Pt-embedded-DNW photodetector can be attributed to four primary contributions: the 1D transport of carriers along the nanowire, the presence of deep-level defects, the LSPR effect generated by Pt nanoparticles, and the efficient carrier separation facilitated by the Pt/diamond Schottky junction. These four contributions jointly improve the DUV responsivity and response speed of the DNW photodetector at high temperatures. Inheriting the intrinsic properties of bulk diamond, such as its high breakdown field strength, thermal stability, and high displacement energy, the nanowire photodetector is expected to remain operational under intense radiation conditions. Therefore, this work highlights the potential of DNW-based devices for DUV detection in harsh environments, advancing their application in fields such as aerospace, industrial monitoring, and defense systems.

## Conclusions

This work demonstrates the fabrication of Pt nanoparticles-embedded DNWs, achieving a highly efficient DUV photodetector under both RT and high-temperature conditions. By employing Pt film deposition, subsequent dewetting, and homoepitaxial CVD growth, uniformly embedded Pt nanoparticles were achieved within smooth DNWs. Remarkably, the Pt-embedded DNWs retain the single-crystal structure of diamond. With oxygen-terminated diamond surface, the Pt-embedded DNW photodetector exhibits a room temperature responsivity of 68.5 A W^−1^ under 220 nm illumination at a bias of 20 V, representing an enhancement of approximately 2000 times compared to oxygen-terminated bulk diamond. The DUV/Visible rejection ratio of the device reaches 550. When the photodetector operates at elevated temperature, the 220 nm responsivity increases gradually and reaches 3098.7 A W^−1^ at 275 °C, maintaining stable and reproducible response characteristics. This exceptional enhancement stems from synergistic mechanisms: the efficient carrier transport provided by the 1D nanowire structure; the role of deep-level traps; the LSPR effect induced by the embedded Pt nanoparticles; and the localized Schottky junctions at the Pt/diamond interface. Together, these factors enhance optical absorption, carrier generation, and separation efficiency. These results demonstrate that Pt-embedded DNWs are highly promising for advanced DUV detection in harsh environments, including applications in aerospace, industrial monitoring, and military applications.

## Supplementary Information

Below is the link to the electronic supplementary material.

Supplementary file1 (DOCX 17946 KB)
