# Supplementary material for: Single-Crystal Diamond Nanowires Embedded with Platinum Nanoparticles for High-Temperature Solar-Blind Photodetector
Source: Nanomicro Lett. 2025 Apr 16;17:220. doi: 10.1007/s40820-025-01746-9 (PMC12003257; doi:10.1007/s40820-025-01746-9)
Supplement: Supplementary file 1 — Supplementary file1 (DOCX 17946 KB) [file 40820_2025_1746_MOESM1_ESM.docx]

Supporting Information for

**Single-Crystal Diamond Nanowires Embedded with Platinum Nanoparticles for High-Temperature Solar-Blind Photodetector**

Jiaqi Lu ^1, 2^, Xinglai Zhang ^1, 2^, Shun Feng ^1, 2^, Bing Yang ^1, 2,^ *, Ming Huang ^3^, Yubin Guo ^1, 2^, Lingyue Weng ^1, 2^, Nan Huang ^1, 2^, Lusheng Liu ^2^, Xin Jiang ^4^, Dongming Sun ^1, 2,^ *, Huiming Cheng ^1, 2, 5^

^1^ School of Materials Science and Engineering, University of Science and Technology of China, Shenyang 110016, P. R. China

^2^ Shenyang National Laboratory for Materials Science, Institute of Metal Research, Chinese Academy of Sciences, Shenyang 110016, P. R. China

^3^ School of Environment and Chemical Engineering, Shenyang University of Technology, Shenyang 110870, P. R. China

^4^ Institute of Materials Engineering, University of Siegen, Siegen, 57076, Germany

^5^ Institute of Technology for Carbon Neutrality, Shenzhen Institute of Advanced Technology, Chinese Academy of Sciences, Shenzhen 518055, P. R. China

*Corresponding authors. E-mail: [byang@imr.ac.cn](mailto:byang@imr.ac.cn) (Bing Yang); [dmsun@imr.ac.cn](mailto:dmsun@imr.ac.cn) (Dongming Sun)

**Supplementary Figures and Tables**


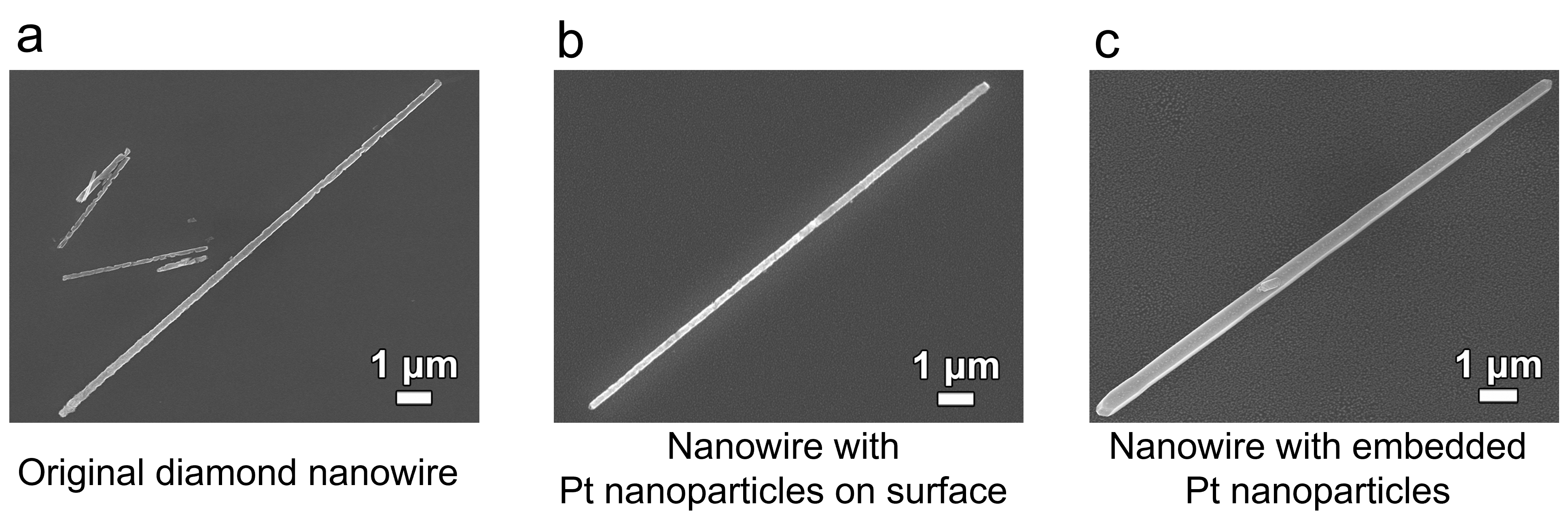


**Fig. S1** Overall SEM images of the original diamond nanowire: after Pt deposition **a** and dewetting **b**, and after homoepitaxy **c**


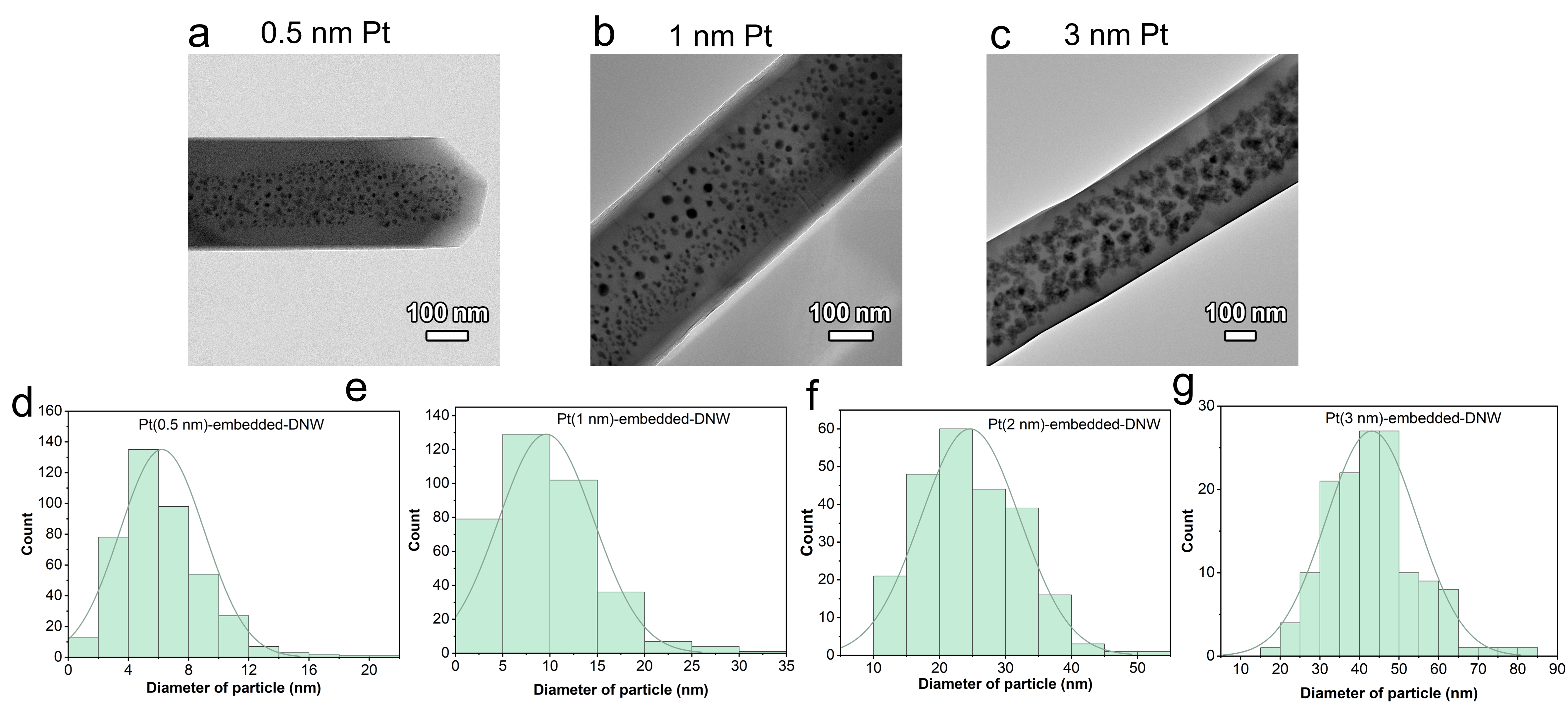


**Fig. S2** **a-c** TEM images of Pt-embedded DNWs corresponding to 0.5 nm, 1 nm, and 3 nm Pt deposition before homoepitaxial process; **d-g** Statistics of Pt nanoparticle sizes in **a**, **b**, Fig. 2a, **c**, respectively


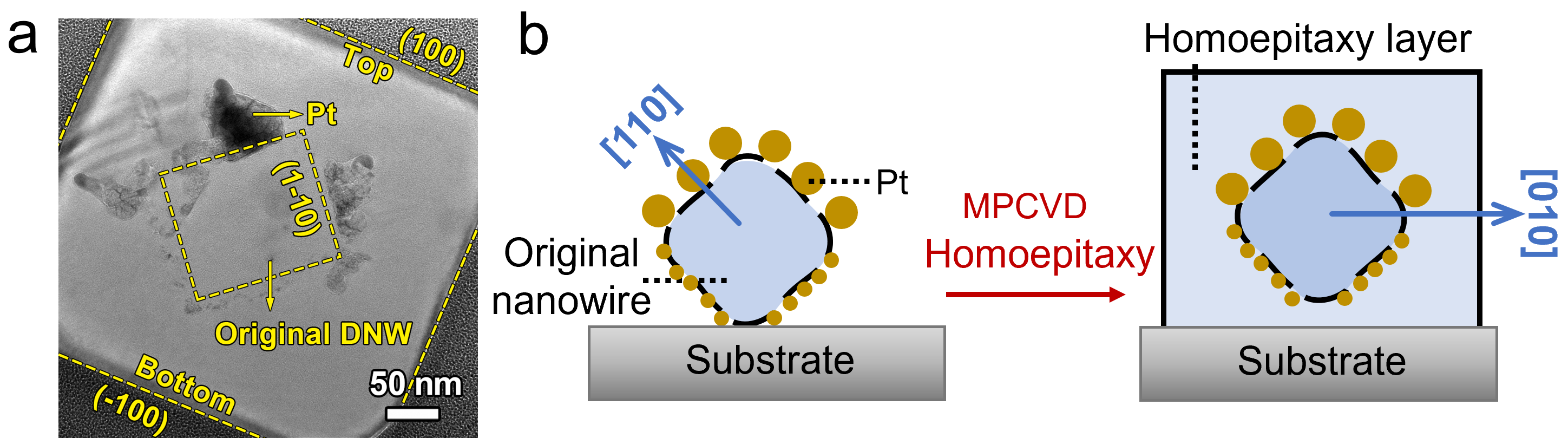


**Fig. S3 a** The cross-sectional TEM image of the Pt-embedded DNW; **b** Schematic diagram mechanism of epitaxial growth of DNW on the quartz substrate surface


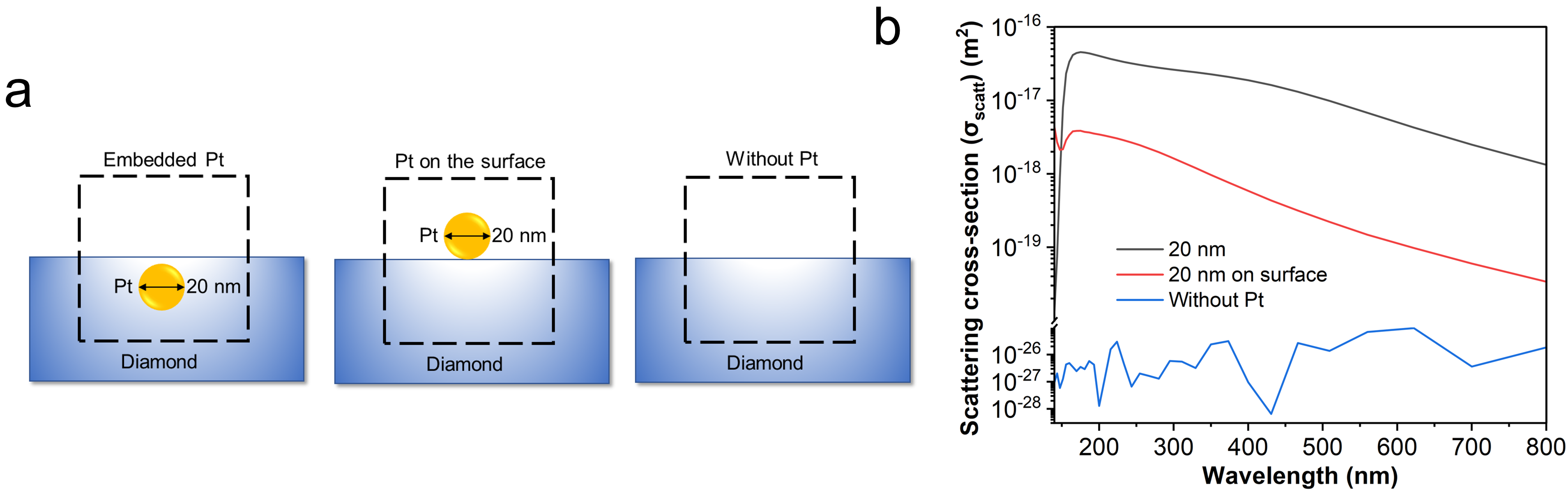


**Fig. S4** Schematic diagram of the model used for finite-difference time-domain (FDTD) simulations **a** and obtained Spectral scattering cross-section **b** corresponding to a 20 nm diameter Pt particle embedded in diamond, on the surface of diamond and absent

In order to explain the LSPR effect, we used finite-difference time-domain (FDTD) simulations to calculate the scattering cross-sections of Pt nanoparticles located on the diamond surface and inside the diamond. The results show that when Pt nanoparticles are embedded within the diamond, they exhibit significantly higher scattering cross-sections. This implies that the Pt embedding structure can lead to strong DUV absorption and photo gain.


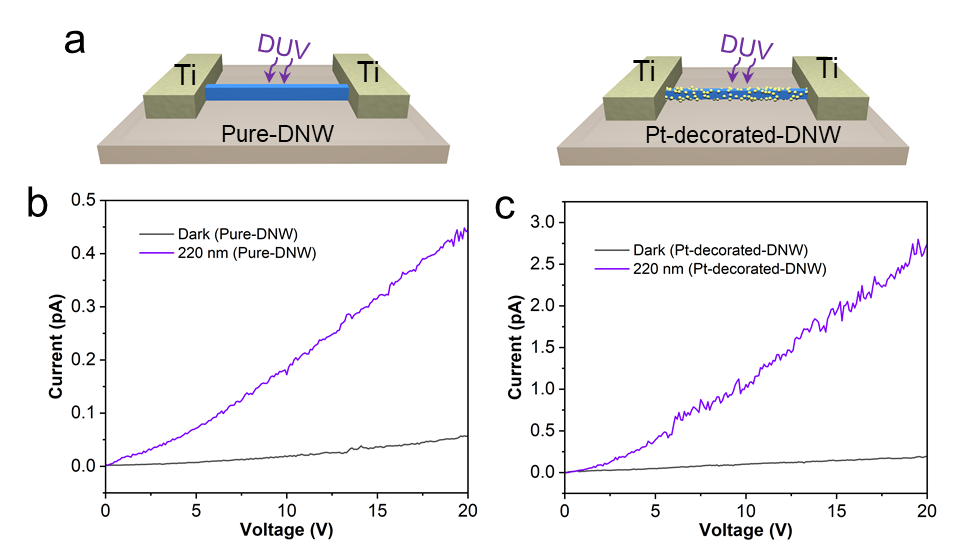


**Fig. S5** **a** Schematic diagrams of DUV photodetectors (Pure-DNW and Pt-decorated-DNW) based on the DNW underwent only homoepitaxial growth and the DNW subjected to homoepitaxial growth followed by Pt nanoparticles decoration; The *I*-*V* curves of DNW photodetectors Pure-DNW **b**, Pt-decorated-DNW **c** under dark and 220 nm DUV light illumination

**Table 1** The effective light area of different devices.

| Device name | Effective light area (m^2^) |
| --- | --- |
| Pure-DNW | 1.95×10^-12^ |
| Pt-decorated-DNW | 2.02×10^-12^ |
| Pt-embedded-DNW | 5.97×10^-12^ |
| Bulk diamond | 2.91×10^-7^ |


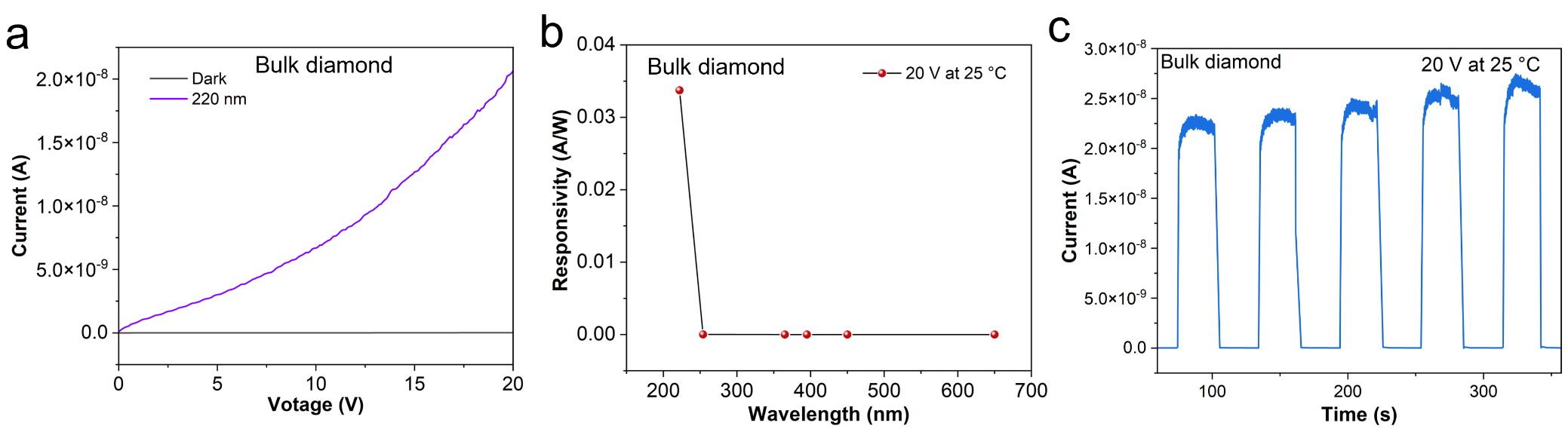


**Fig. S6 a** *I*-*V* curves of the photodetector based on bulk single-crystal diamond under 220-nm DUV light illumination and dark; **b** and **c** Spectral response and time dependent response of the bulk single-crystal diamond under a bias of 20 V


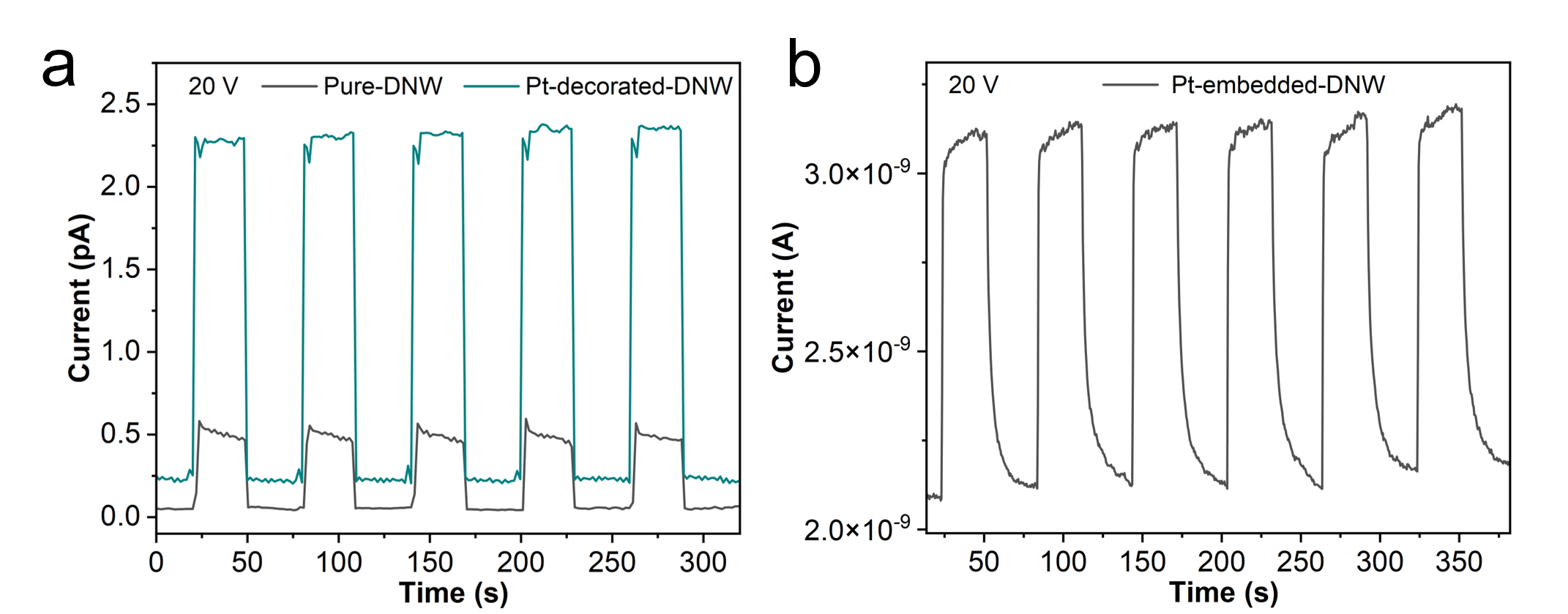


**Fig. S7** **a** Time dependent photoresponses of the Pure-DNW and Pt-decorated-DNW under the 220 nm light at a bias of 20 V; **b** Time dependent photoresponses of the Pt-embedded-DNW under the 220 nm light at a bias of 20 V


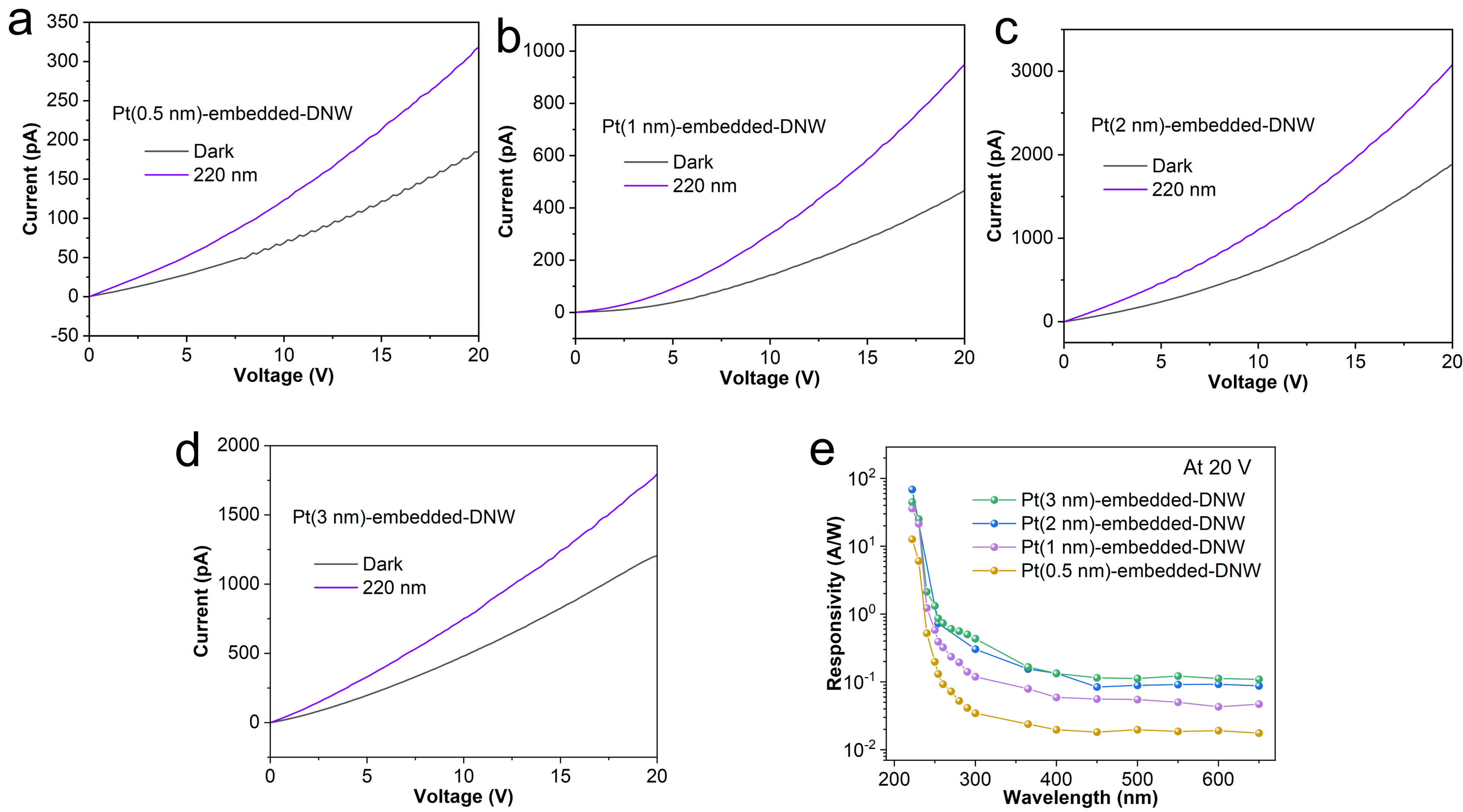


**Fig. S8** *I*-*V* curves of DNW photodetectors Pt(0.5 nm)-embedded-DNW **a**, Pt(1 nm)-embedded-DNW **b**, Pt(2 nm)-embedded-DNW **c**, Pt(3 nm)-embedded-DNW **d** under dark and 220 nm DUV light illumination. **e** Spectral responsivity of the four photodetectors under a bias of 20 V


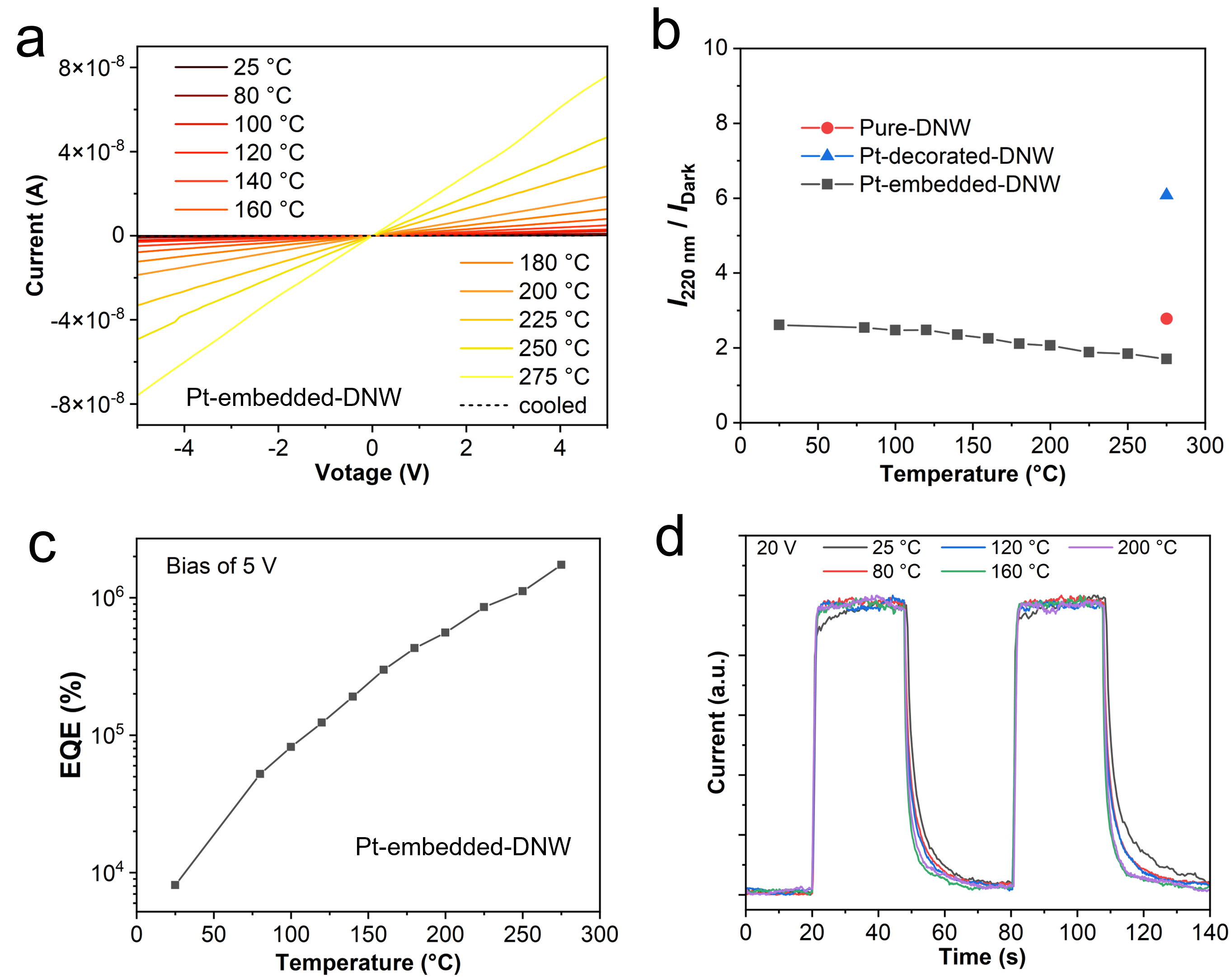


**Fig. S9** Photo response under 220 nm illumination at various temperature ranged from 25 °C to 275 °C: **a** Dark *I*-*V* curve of Pt-embedded-DNW; **b** Variation of On-Off (*I*_220 nm_/*I*_Dark_) ratio with temperature of different photodetectors; **c** The trend of external quantum efficiency (EQE) for the 220 nm light with temperature of Pt-embedded-DNW; **d** The time dependent response of the Pt-embedded-DNW to 220 nm light at different temperatures

Figure S9c illustrates the time-resolved photoresponses of the Pt-embedded-DNW device under 220 nm light at a bias of 20 V at different temperatures. The decay time *t*_decay_ of the Pt-embedded-DNW decreases from 9.01 s at RT to 7.09 s at 200 °C.


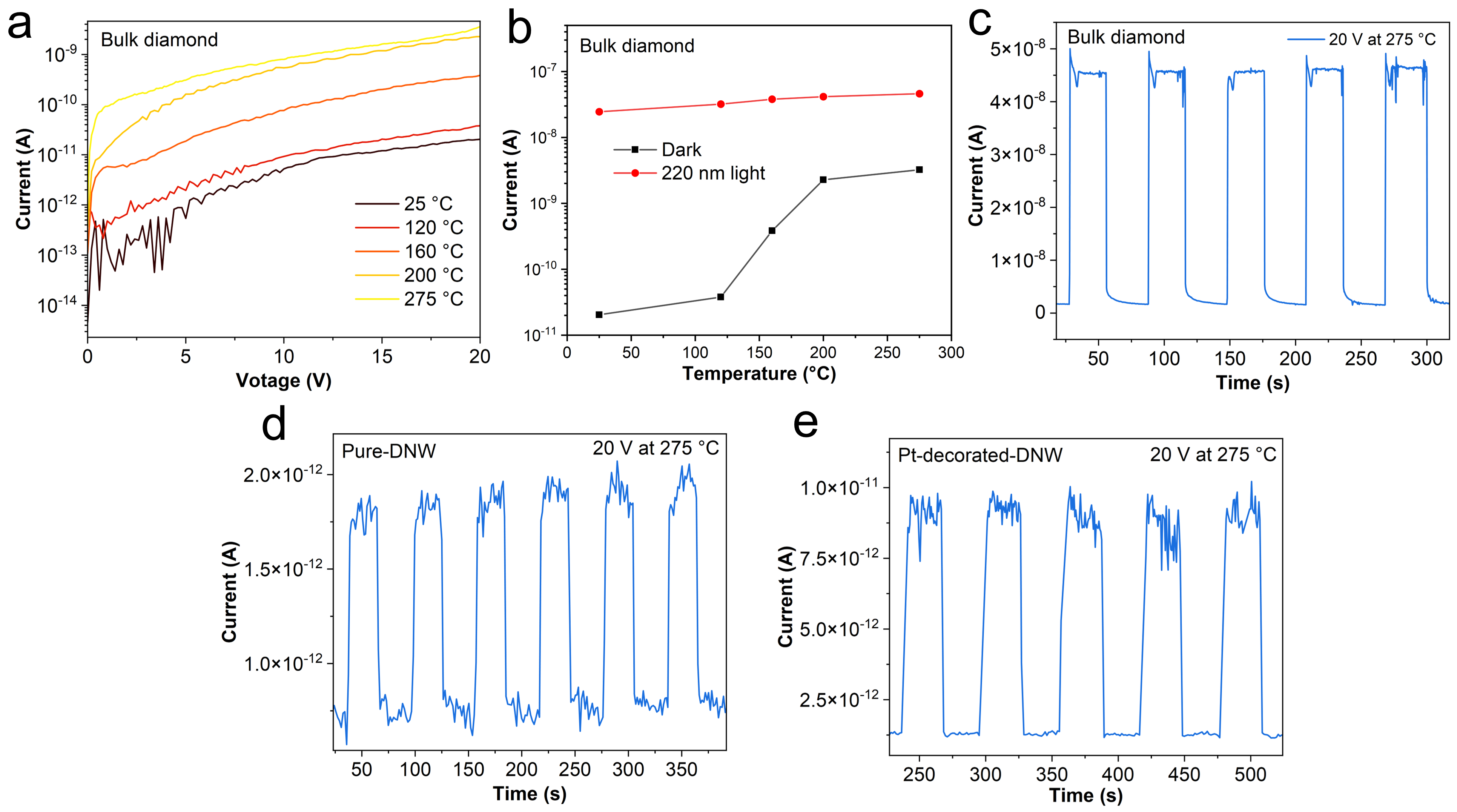


**Fig. S10 a** Dark *I*-*V* curve of bulk diamond; **b** Variation of dark current and photocurrent with temperature at a bias of 20 V of bulk diamond; Time dependent photoresponses of the Bulk diamond **c**, Pure-DNW **d**, and Pt-decorated-DNW **e** under the 220 nm light at a bias of 20 V and 275 °C


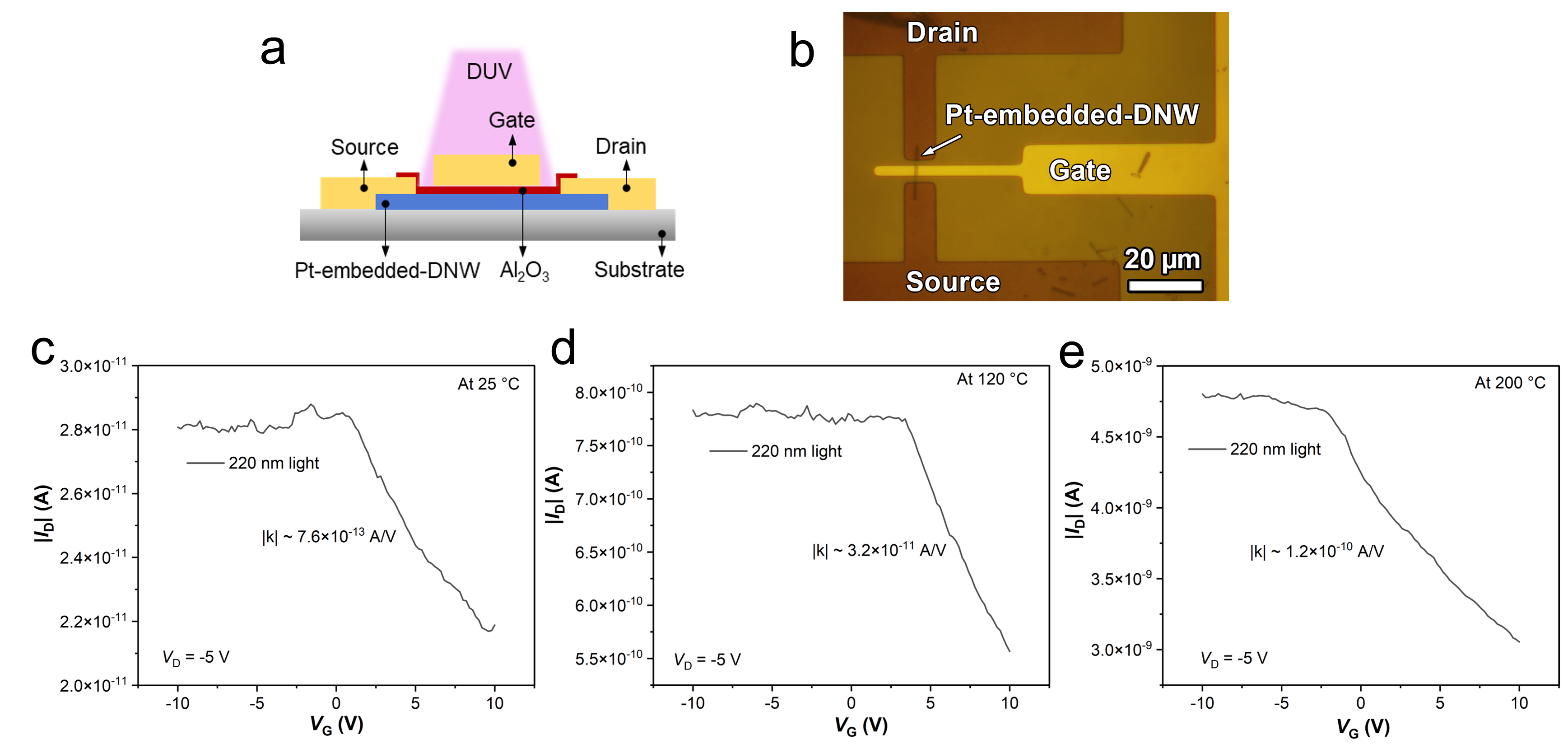


**Fig. S11 a-b** Schematic and actual optical microscope image of the field-effect transistor (FET) based on Pt-dembedded DNW; **c-e** The transfer characteristic curves under 220 nm light illumination of the FET at RT, 120 °C, and 200 °C

As show in Fig. S11a-b, a field-effect transistor (FET) based on Pt-dembedded DNW was fabricated to measure the mobility at different temperatures. The gate of transistor cannot work in the dark state owing to low carrier concentration inside the nanowire. When the 220 nm light is illuminated and no gate bias (*V*_G_) is applied, the drain current (*I*_D_) exhibits an increase, similar to the two-end photodetector in Fig. 4. The drain current decreases with more positive gate bias at RT, 120 °C, and 200 °C, as displayed in the transfer characteristic curves in Fig. S11c-e. For the FET, the carrier mobility (*μ*_FET_) can be calculated using the following equation:

$\mu_{FET}=\frac{L}{WC_{i}V_{D}}\cdot\frac{\partial I_{D}}{\partial V_{G}}$ (S1)

where *L* is the gate length, *W* is the gate width, *C_i_* is the dielectric layer capacitance, and *V*_D_ is the drain bias. It can be noted that the carrier mobility is positively correlated with the slope (k) of the transfer characteristics curves. The calculated slopes in Fig. S11c-e increase with rising temperature, implying that carrier mobility exhibit an increase trend with temperature.


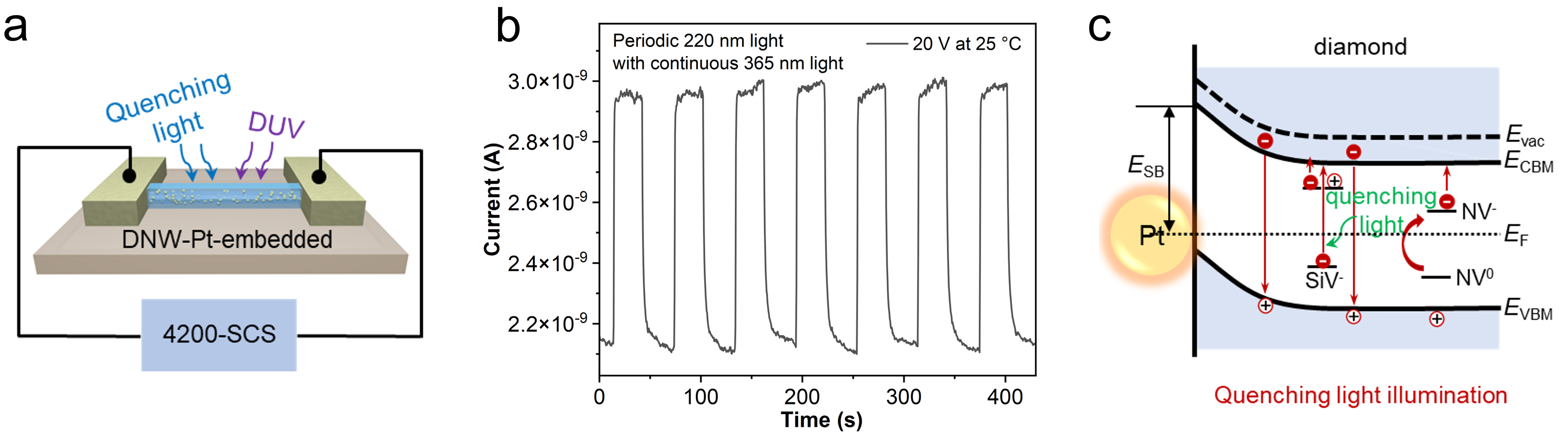


**Fig. S12** Schematic **a** and dynamic current response **b** of Pt-embedded-DNW when it is irradiated by periodic 220 nm DUV light and continuous 365 nm quenching light under a bias of 20 V; **c** Energy band diagram of the embedded Pt/diamond interface within nanowire under quenching light illumination


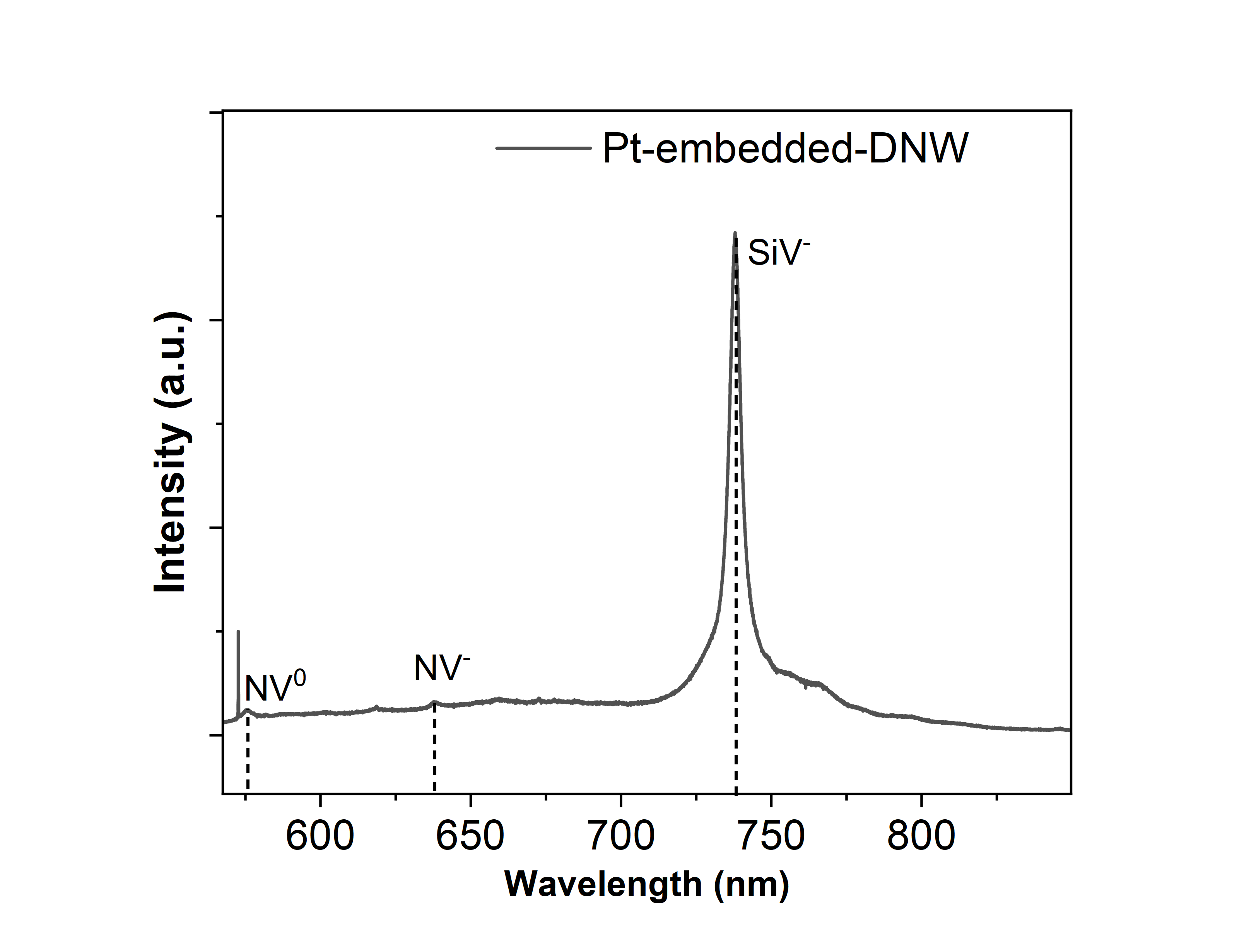


**Fig. S13** The photoluminescence spectrum of the Pt-embedded-DNW

Figure S12 shows the photoluminescence (PL) spectrum of the Pt-embedded-DNW. The PL peak of NV^0^, NV^-^, and SiV^-^ can be identified at 575 nm, 637 nm and 738 nm, respectively.


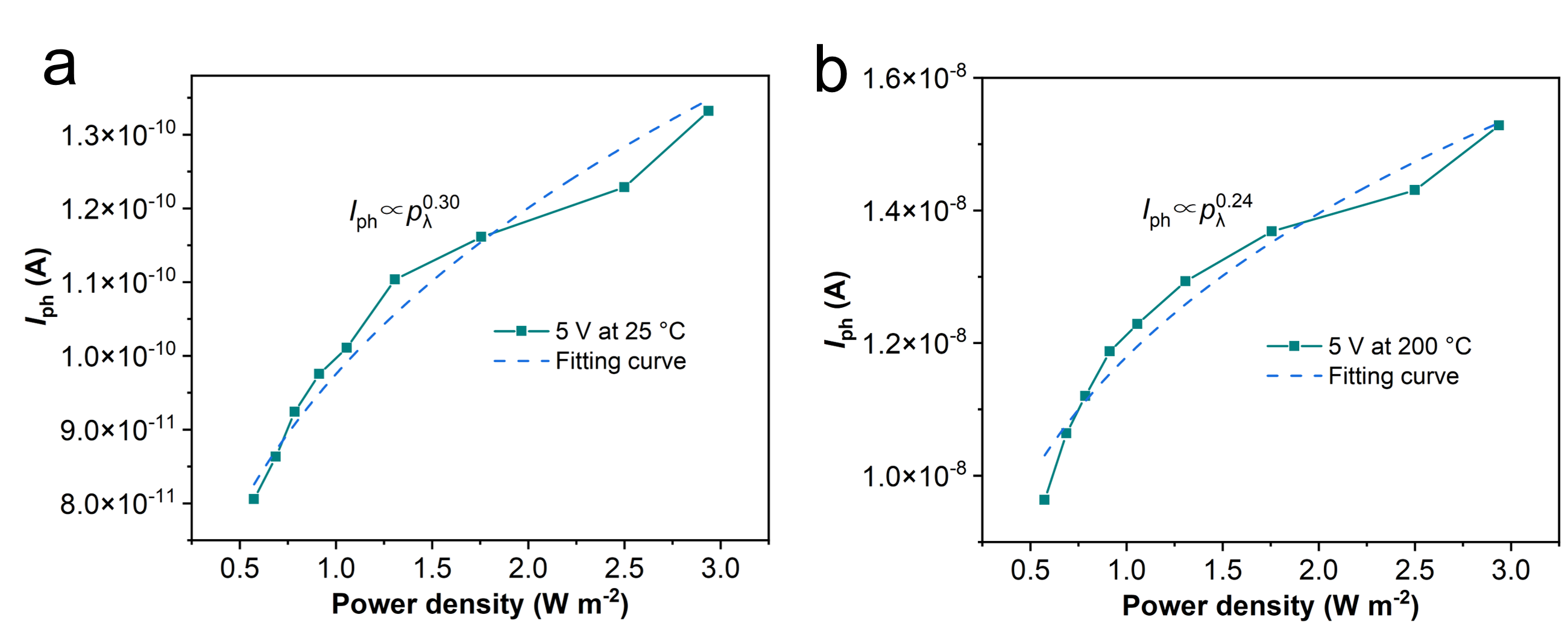


**Fig. S14** The plot of net photocurrents with the illumination power at a fixed incident-light wavelength of 220 nm at 25 °C **a** and 200 °C **b** of Pt-embedded-DNW


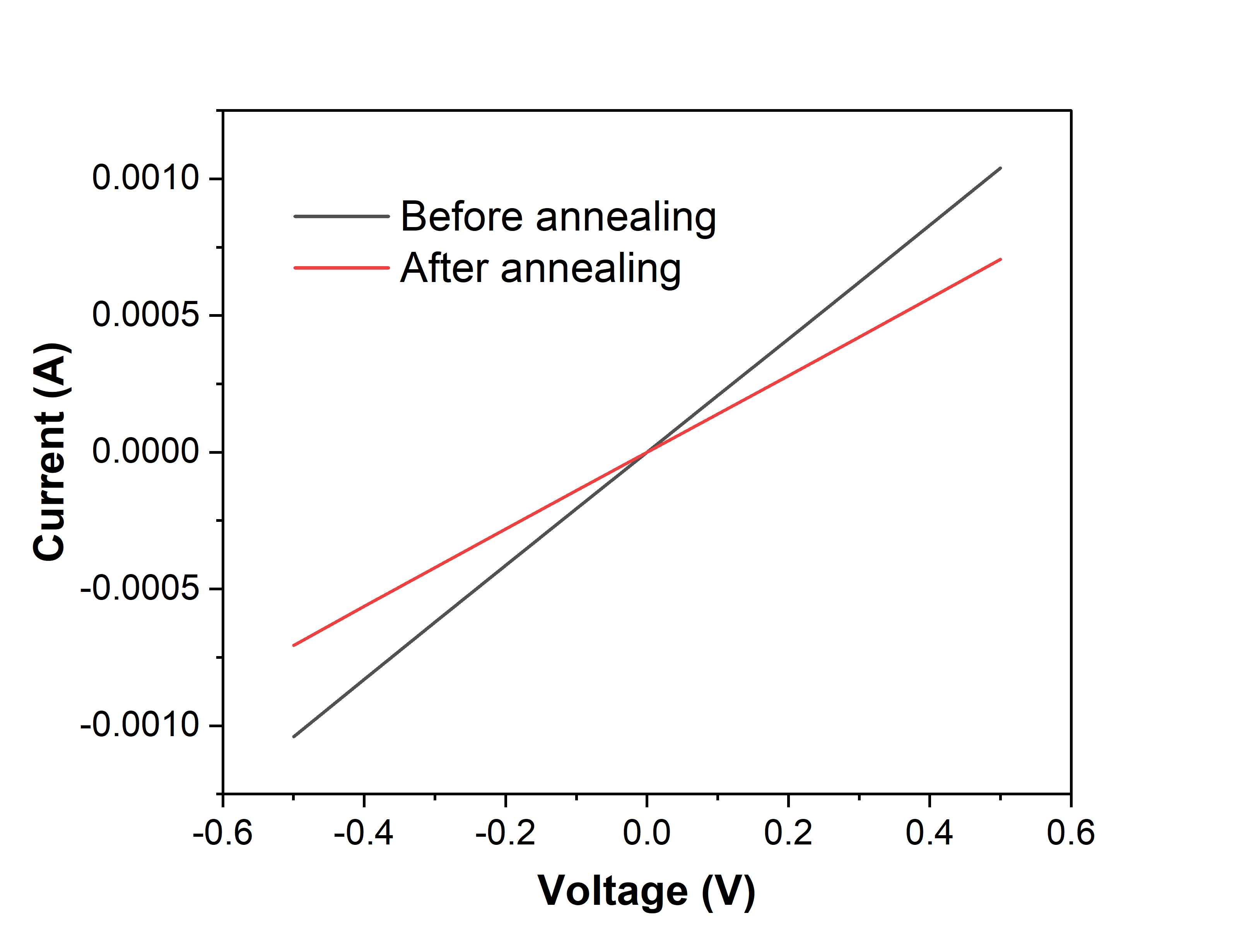


**Fig. S15** *I*-*V* curves of a 2×4 mm^2^ Ti electrode on a quartz substrat before and after annealing

To check the conductivity of the Ti electrode after annealing, a 1×3 mm2 Ti electrode was fabricated on quartz substrate. As shown in Fig. S15, the I-V curves were measured using a semiconductor analyzer by contacting probes to the two ends of the electrode before and after annealing treatment. The measured resistances were 482 Ω and 708 Ω before and after annealing, respectively, indicating a slight increase in resistance caused by the formation of a thin oxide layer on the Ti surface. However, the device operation temperature is below 300 °C. This suggests that the Ti electrodes will not be degraded and can be used to maintain good device performance.


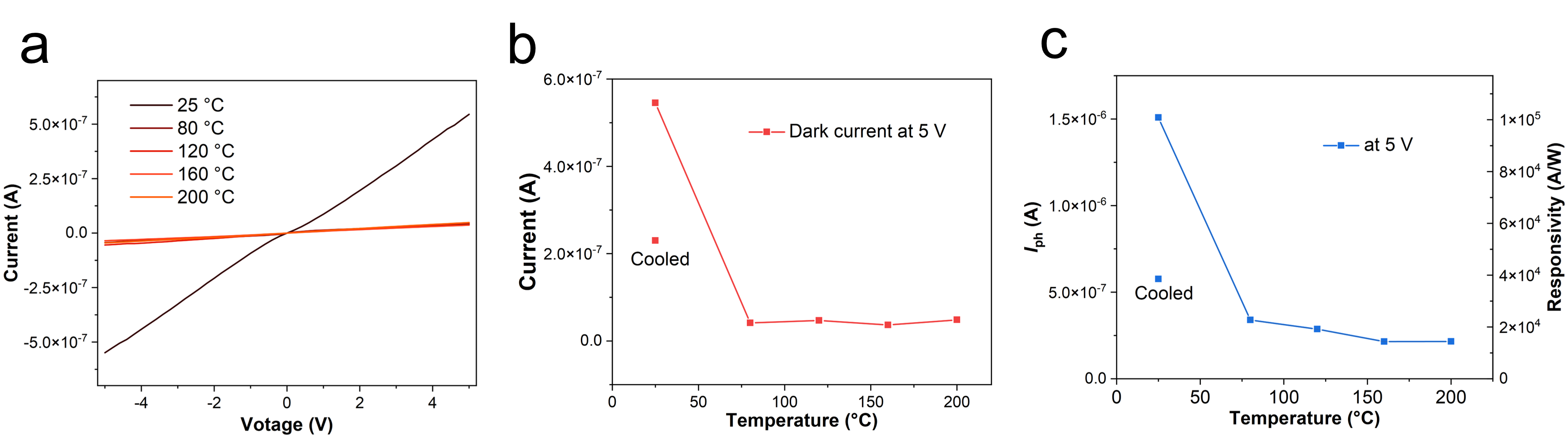


**Fig. S16** **a** Dark current of the hydrogen-terminated DNW photodetector at different temperatures; Variation in Dark current **b** and net photocurrent **c** with temperature at a 5 V bias

The hydrogen-terminated DNW photodetectors exhibited high dark current and high photocurrent at low bias at room temperature. The responsivity was about 1.01×10^5^ A/W. However, both the dark current and the photocurrent decreased significantly as the temperature increases (Fig. S16). The responsivity decreased to 1.44×10^4^ A/W at 275 °C. And the device's performance does not recover quickly to its pre-heated state after cooling. Therefore, the hydrogen-terminated DNW exhibited instable performance or even degradation during temperature fluctuations, compared with the Pt-embedded-DNW.
